# A Quantitative Analysis of Electrochemical CO_2_ Reduction on Copper in Organic Amide and Nitrile-Based Electrolytes

**DOI:** 10.1021/acs.jpcc.3c01955

**Published:** 2023-07-03

**Authors:** Asvin Sajeev Kumar, Marilia Pupo, Kostadin V. Petrov, Mahinder Ramdin, J. Ruud van Ommen, Wiebren de Jong, Ruud Kortlever

**Affiliations:** † Department of Process & Energy, Faculty of Mechanical, Maritime & Materials Engineering, Delft University of Technology, Leeghwaterstraat 39, 2628 CB Delft, The Netherlands; ‡ Department of Chemical Engineering, Faculty of Applied Sciences, Delft University of Technology, Van der Maasweg 9, 2629 HZ Delft, The Netherlands

## Abstract

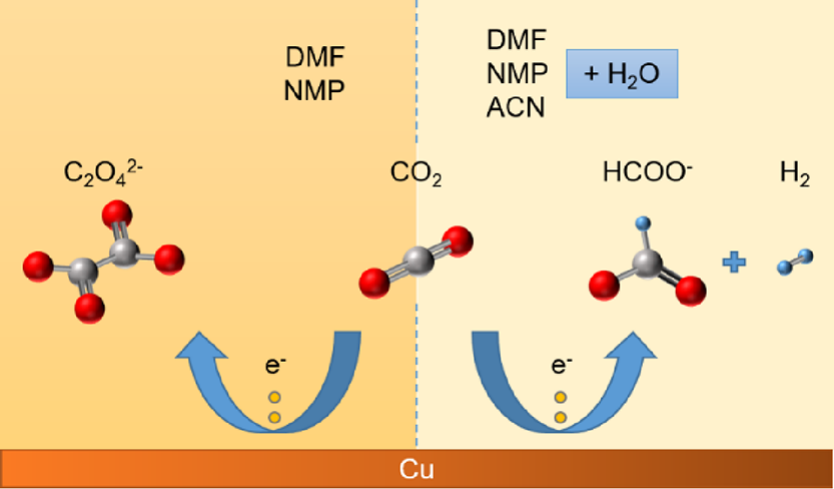

Aqueous electrolytes used in CO_2_ electroreduction typically have a CO_2_ solubility of around 34 mM under ambient conditions, contributing to mass transfer limitations in the system. Non-aqueous electrolytes exhibit higher CO_2_ solubility (by 5–8-fold) and also provide possibilities to suppress the undesired hydrogen evolution reaction (HER). On the other hand, a proton donor is needed to produce many of the products commonly obtained with aqueous electrolytes. This work investigates the electrochemical CO_2_ reduction performance of copper in non-aqueous electrolytes based on dimethylformamide (DMF), *n*-methyl-2-pyrrolidone (NMP), and acetonitrile (ACN). The main objective is to analyze whether non-aqueous electrolytes are a viable alternative to aqueous electrolytes for hydrocarbon production. Additionally, the effects of aqueous/non-aqueous anolytes, membrane, and the selection of a potential window on the electrochemical CO_2_ reduction performance are addressed in this study. Experiments with pure DMF and NMP mainly produced oxalate with a faradaic efficiency (FE) reaching >80%; however, pure ACN mainly produced hydrogen and formate due to the presence of more residual water in the system. Addition of 5% (v/v) water to the non-aqueous electrolytes resulted in increased HER and formate production with negligible hydrocarbon production. Hence, we conclude that aqueous electrolytes remain a better choice for the production of hydrocarbons and alcohols on a copper electrode, while organic electrolytes based on DMF and NMP can be used to obtain a high selectivity toward oxalate and formate.

## 1 Introduction

The continued usage of fossil fuels and feedstock has led to a rise in atmospheric CO_2_ concentrations, accelerating global warming and endangering the global ecological balance.^1^ Among the various mitigation strategies such as CO_2_ capture coupled with either CO_2_ storage or CO_2_ conversion technologies, CO_2_ conversion has been gaining increasing attention recently owing to its capability to convert CO_2_ into value-added molecules that can be used as fuels or feedstock for the chemical industry.^1,2^

CO_2_ conversion can be achieved via various routes including thermochemical, photochemical, electrochemical, and photoelectrochemical routes.^2^ Among these, the electrochemical reduction of CO_2_ is promising as it can operate in mild conditions and utilize renewable energy sources such as wind and solar.^2,3^ Some of the other benefits of electrochemical CO_2_ reduction include the ability to control the reaction rates by adjusting the external applied potential and the production of a variety of C_1_–C_3_ gaseous and liquid products.^4−7^ Despite all these promising features, electrochemical CO_2_ reduction is still far from being commercialized. This is mainly due to the high overpotentials required for product formation, limited product selectivity, and relatively low current densities.^3^ For example, typical current densities achieved in liquid-phase CO_2_ reduction are in the order of mA/cm^2^ of electrode surface while industrial water electrolyzers are typically operated at current densities in the order of A/cm^2^.^8,9^

Mass transfer limitations are a main concern in CO_2_ reduction cells as these result in reduced product selectivities and current densities.^10,11^ The limited dissolution of CO_2_ into aqueous electrolytes and slow diffusion of dissolved CO_2_ from the bulk of the electrolyte to the surface of the working electrode, where it is instantly consumed, make the mass transfer of CO_2_ the rate-limiting step. Diffusion limitations can be minimized by altering cell designs or by choosing an electrolyte in which more CO_2_ can be dissolved, making more CO_2_ available for reaction.

Aqueous electrolytes are the most commonly employed electrolytes in the electrochemical CO_2_ reduction reaction (CO_2_RR). However, the overall solubility of CO_2_ in water is only around 34 mM in ambient conditions, contributing to significant mass transfer limitations in the system.^12^ In an attempt to improve the overall efficiency and selectivity of CO_2_RR, several studies have experimented with various non-aqueous electrolytes.^13−16^ Non-aqueous electrolytes generally exhibit higher CO_2_ solubility (by five to eight-fold compared to aqueous electrolytes), making them attractive alternatives to aqueous systems. Non-aqueous electrolytes also provide additional possibilities to suppress the undesirable hydrogen evolution reaction (HER) by limiting the proton availability near the catalytic surface. As protons are also required to form certain CO_2_RR products, the reaction pathways in non-aqueous electrolytes can differ from those occurring in aqueous electrolytes. Overall, using non-aqueous electrolytes can enable better CO_2_RR product selectivity and lead to the production of different products.^17−19^ As these electrolytes are generally stable, the detected products seldom contain carbon atoms that stem from the organic electrolytes, apart from those coming from the supplied CO_2_.^15,20^ Additionally, it has been shown that mixing electrolytes, such as the addition of water to non-aqueous electrolytes, significantly affects the product distribution and product selectivity.^21,22^

Several studies have reported the use of organic electrolytes for electrochemical CO_2_ reduction on Au to improve the selectivity toward CO by suppressing HER.^16,23,24^ However, the use of Cu electrodes with non-aqueous electrolytes is hardly reported.^12,25^ Cu is the only electrocatalytic material able to reduce CO_2_ into various C_2+_ products in aqueous electrolytes with decent selectivities, making it one of the most interesting CO_2_RR electrocatalysts.^26−30^ Also, previous studies involving non-aqueous electrolytes have been predominantly qualitative in nature with limited focus on the quantification of the products.^15,16,21,31^ Hence, in this study, we investigate the electrochemical CO_2_ reduction performance of Cu in non-aqueous organic electrolytes based on dimethylformamide (DMF), *n*-methyl-2-pyrrolidone (NMP), and acetonitrile (ACN) with a focus on the products formed during the reaction. The results of CO_2_ reduction in non-aqueous electrolytes are also compared with that in the standard 0.1 M aqueous KHCO_3_ electrolyte to analyze if the non-aqueous electrolytes could outperform the aqueous electrolytes in terms of hydrocarbon production. In addition, we study the effect of water addition to the above electrolytes to explore the possibility of producing hydrocarbons in electrolytes with limited proton availability.

## 2 Experiments

### 2.1 Materials

The aqueous electrolytes are prepared from KHCO_3_ (≥99.95% trace metals basis, Sigma-Aldrich) and ultrapure water (Milli-Q, 18.2 MΩ cm, 1.5 ppb TOC). The non-aqueous electrolytes are prepared from DMF (anhydrous, 99.8%, <0.005% water, Sigma-Aldrich), NMP (anhydrous, 99.5%, <0.005% water, Sigma-Aldrich), ACN (anhydrous, 99.8%, <0.005% water, Sigma-Aldrich), and tetrabutylammonium hexafluorophosphate salt (TBAPF_6_ for electrochemical analysis, ≥99.0%, Sigma-Aldrich). All the chemicals (electrolytes and salt) are used as received. The non-aqueous electrolyte–water mixtures are prepared by adding the required amounts of ultrapure water to the non-aqueous electrolytes. All solutions are prepared under ambient conditions.

### 2.2 Electrode Preparation

Copper (Cu) foil (99.999% trace metals basis, Sigma-Aldrich) and platinum (Pt) foil (99.9% trace metals basis, Sigma-Aldrich) are used as the working and counter electrodes, respectively, in all experiments. The newly purchased Cu foil is initially sanded using sandpaper of different grades (P320, P800, P1200, and P2000, Struers) and mechanically polished (3 and 1 μm diamond suspension, Struers) to provide a mirror-like finish to the Cu electrode surface. The polished electrode is then ultrasonicated in isopropanol for 15 min to remove any impurities from the polishing procedure. Afterward, the Cu electrode is electropolished in phosphoric acid (BioUltra, ≥85% (T), Sigma-Aldrich), using the Cu electrode as the working electrode and a carbon rod as the counter and reference electrode. An oxidation potential of 2.1 V (vs counter reference) is applied to the Cu electrode for 3 min using a multichannel potentiostat (VSP-300, Biologic) for the electropolishing process. The Cu electrode is then cleaned with ultrapure water and purged with N_2_ (99.999%, Linde) for 30 s to remove any moisture from the surface. The Pt counter electrode is first rinsed with ultrapure water and flame-annealed to oxidize any organic impurities on the metal surface before its introduction into the electrochemical cell. The electropolishing of Cu and flame annealing of Pt is repeated before each experiment.

### 2.3 Electrochemical Experiments

The electrochemical experiments are performed in a small H-cell with continuous gas flow, similar to the cell designed by Lobaccaro et al.,^32^ with an electrolyte capacity of 1.8 mL and an exposed electrode (geometric) surface area of 1 cm^2^ at both the cathode and anode sides. A schematic of the cell geometry and the experimental setup is shown in Figure S1. A Selemion AMVN (AGC Chemicals) anion exchange membrane is used to separate the two compartments while maintaining the ion conductivity, in the case of aqueous electrolytes. In the case of non-aqueous electrolytes, a Nafion 117 (Ion Power) cation exchange membrane is used. CO_2_ gas (99.999%, Linde) is bubbled through the catholyte solution for 20 min before the start of the experiments and continued during experimental runs at 8 mL_n_/min flow rate. All electrochemical experiments are performed using a leak-free Ag/AgCl reference electrode (LF-1-45, Alvatek) and a single-channel potentiostat (SP-200, Biologic). The cyclic voltammetry measurements are performed over a potential range of 0 to −2.5 V (vs Ag/AgCl) in still conditions (without gas flow) using electrolyte pre-saturated with Ar (99.999%, Linde) or CO_2_. The anode chamber is left open to the atmosphere in all experiments. All potentials in this study are reported against Ag/AgCl, unless otherwise specified.

### 2.4 Product Analysis

The CO_2_ flow to the inlet of the cell is controlled using a mass flow controller (EL-FLOW Select, Bronkhorst), and the flow of gas at the outlet of the cell is measured using a mass flow meter (EL-FLOW Select, Bronkhorst). The outlet gas from the cathode chamber is injected every 2 min into a gas chromatograph (GC, CompactGC 4.0, Interscience) to analyze the amount of gaseous products. The GC has one channel with a flame ionization detector (FID) for analyzing C_1_–C_6_ gases and two channels with thermal conductivity detectors (TCD) for analyzing CO and H_2_, respectively. The FID channel consists of an Rtx-1, 5.00 μm (15 m × 0.32 mm) analytical column, the first TCD channel consists of a Carboxen 1010 (3 m × 0.32 mm) pre-column and a Molsieve 5A (5 m × 0.32 mm) analytical column, and the second TCD channel consists of a Carboxen 1010 (3 m × 0.32 mm) pre-column and a Molsieve 5A (7 m × 0.32 mm) analytical column for the separation of the components before entering the respective channel detectors. Aliquots of the catholyte and anolyte are collected at the end of each experiment and analyzed using a high-performance liquid chromatography (HPLC, 1290 Infinity II, Agilent) to determine the amount of liquid products. Two Aminex HPX-87H (Biorad) organic acid analysis columns placed in series are used for the separation of the components before entering the refractive index detector (RID) in the HPLC. Due to the overlapping retention times/chemical shifts of DMF and formate in the HPLC/NMR (nuclear magnetic resonance) spectroscopy, an ion chromatograph (881 Compact IC Pro, Metrohm) fitted with a Metrosep A Supp 5-150/4.0 column is used for the quantification of formate in the case of DMF. The actual water content of the non-aqueous electrolytes is measured using Karl Fischer Coulometry (756 KF Coulometer, Mettler Toledo).

## 3 Results and Discussion

### 3.1 Electrolyte Composition

Tetraethylammonium (TEA) and tetrabutylammonium (TBA) are the most commonly used cations in electrochemical experiments with organic electrolytes as these tetra-alkylammonium (R_4_N^+^) ions have good solubility and are inert under reductive conditions.^25,33−38^ These cations are commonly used in combination with perchlorate, tetrafluoroborate, or hexafluorophosphate anions. Earlier experiments have been performed predominantly with perchlorate salts; however, as perchlorates pose explosion hazard concerns, their usage has declined. Tetrafluoroborate and hexafluorophosphate salts are excellent alternatives to perchlorates,^39−41^ and hence in this study, all experiments are performed with tetrabutylammonium hexafluorophosphate (TBAPF_6_).

Solubility tests performed with 0.1 M TBAPF_6_ salt in DMF, NMP, and ACN with varying amounts of water show that the salt is soluble in dilutions with more than 80% (v/v) of the organic electrolyte (Table S1). Earlier work by Figueiredo et al.^42^ has shown that even trace amounts of water (≥46 ppm) can impact the activity and selectivity of CO_2_RR in aprotic electrolytes, while an H_2_O/ACN molar ratio of around 0.25 yielded the highest activity toward CO_2_ reduction with nanostructured Cu electrodes.^31^ In the case of DMF and NMP, a mixture of 95% (v/v) organic electrolyte and 5% (v/v) water gives an H_2_O/electrolyte molar ratio of 0.23 and 0.28, respectively. Hence, the electrochemical performance of 95% (v/v) mixtures of the non-aqueous electrolytes with water are studied alongside the pure electrolytes.

### 3.2 Experimental Design

**Figure 1:**
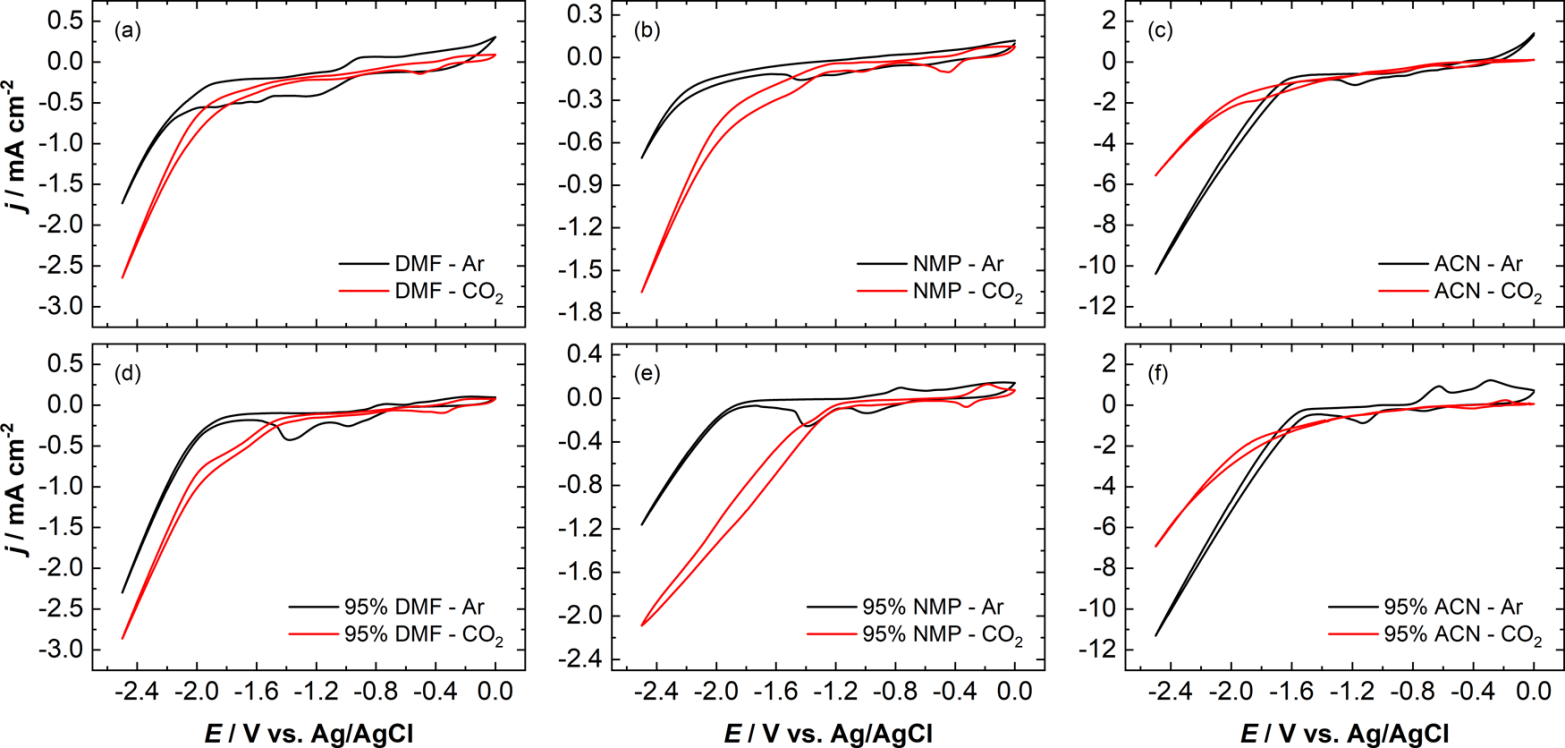
Cyclic voltammograms of pure and 95% (v/v) mixtures of DMF, NMP, and ACN electrolytes with 0.1 M TBAPF_6_ salt in Ar-saturated and CO_2_-saturated conditions, recorded at a scan rate of 20 mV s^–1^.

Cyclic voltammetry (CV) experiments are performed to determine the potential windows of the pure and 95% (v/v) mixtures of DMF, NMP, and ACN electrolytes with 0.1 M TBAPF_6_ salt, in Ar-saturated and CO_2_-saturated conditions. The corresponding voltammograms are shown in Figure 1.

The CVs performed in pure DMF, NMP, and ACN electrolytes under Ar-saturated conditions (Figure 1a–c) show a sharp increase in the reduction current at around −1.9, −1.8, and −1.7 V, respectively. However, under CO_2_-saturated conditions, the increase in reduction current can be observed at two potentials, i.e., −1.5 and −1.9 V for DMF, −1.3 and −1.8 V for NMP, and −1.2 and −1.7 V for ACN (see Table S2 for the summary of potentials). The second reduction potentials observed under CO_2_-saturated conditions also correspond to the reduction potentials observed under Ar-saturated conditions for all three electrolytes. This suggests that this is the onset potential for the reduction of the electrolyte or the supporting electrolyte salt and not the reduction of CO_2_. The first reduction potentials observed at around −1.5, −1.3, and −1.2 V for DMF, NMP, and ACN, respectively, are denoted as the onset potentials for CO_2_ reduction since these reduction features are only observed in CO_2_-saturated conditions.

The cathodic peaks observed around −0.4 and −0.8 V in the case of 95% (v/v) ACN (Figure 1f) are attributed to the reduction of Cu(II) to Cu(I) and Cu(I) to Cu(0), respectively, while the anodic peaks observed around −0.6 and −0.2 V are attributed to the formation of a first layer of Cu(I) oxide and the formation of a second layer consisting of a mixture of Cu(II) oxide and hydroxide, respectively. Similar studies on copper in aqueous solutions and acetonitrile–water mixtures have confirmed the formation of these copper oxide layers.^31,43−45^ Furthermore, hydrogen electro-adsorption on noble metals in underpotential conditions has been observed before with similar voltammetry profiles in aqueous alkaline media and acetonitrile for low-indexed noble metal electrodes.^31,43,46^ Hence, the cathodic peaks observed around −1.2 V are attributed to the hydrogen electro-adsorption, and similar peaks observed in the cases of 95% (v/v) dilutions of NMP and DMF are also attributed to the same phenomena. The current densities obtained in the cases with 95% (v/v) dilutions of DMF, NMP, and ACN (Figure 1d–f) are slightly higher than that obtained with the respective pure electrolytes (Figure 1a–c) due to a drop in the solution resistance with the addition of water.^47^ A slightly positive shift in onset potentials is observed in the cases with NMP and ACN, similar to the results obtained by Joshi et al.^47^ However, this shift is marginal in the case of DMF.

Previous studies have suggested that the onset potentials for CO_2_ reduction in DMF and ACN are around −2.0 and −1.7 V, respectively, at boron-doped diamond or gold electrodes with 0.1 M TBA-based supporting electrolyte salts.^15,16^ However, the electrochemical performance and product distribution obtained with the electrolyte have not been investigated thoroughly to clearly understand if the mentioned potentials are the true onset potentials for CO_2_ reduction in these electrolytes. To verify the CO_2_ reduction onset potentials and to select the optimal configuration, two different experimental configurations are compared. The first configuration is similar to that used in the aqueous systems, where the catholyte and anolyte compartments are filled with the same electrolyte and the compartments are separated by a Selemion anion exchange membrane.^32,48^ The second configuration is similar to that used more often in studies investigating non-aqueous systems, where the catholyte is a non-aqueous electrolyte and the anolyte is an (acidic) aqueous electrolyte, separated by a Nafion cation exchange membrane.^16,19,49,50^ Electrochemical experiments are initially performed with 0.1 M TBAPF_6_ in DMF considering the second reduction potential (−1.9 V) as the onset potential for CO_2_ reduction, as this is very close to the typical onset potential reported in the literature.^15,16,25^Figure S2 shows the faradaic efficiencies (FE) of all the gaseous products and the current densities obtained at applied potentials of −1.9, −2.1, and −2.3 V using the first experimental configuration. CO and H_2_ are the main gaseous products obtained over this potential range with the FE toward CO peaking at 52% at −2.1 V and decreasing to 42% at −2.3 V. The FE toward H_2_ decreased from 16% to 2% as the applied potential is increased from −1.9 to −2.3 V. The production of H_2_ in non-aqueous electrolytes mainly stems from the reduction of residual water present in the system; however, it could also be produced from the reduction of the organic electrolyte or the electrolyte salt.^18,51^ The liquid products typically expected in the case of non-aqueous electrolytes are oxalate and formate, where oxalate is mainly produced in the absence of water and formate is mainly produced in the presence of water.^18,19,25,52^ In the experiments performed, the FE toward oxalate is observed to be abnormally high, adding to more than 80%, leading to a total FE of 130–150% at all the three potentials. This strongly suggests that (a part of) the observed products are formed from the reduction or breakdown of the electrolyte and not from the supplied CO_2_.^12,20^ Oxalate is also detected in the anolyte, which is due to the migration of the oxalate anions through the anion exchange membrane in the first configuration. Therefore, the second (more negative potential) reduction peaks are considered as electrolyte breakdown peaks.

**Figure 2:**
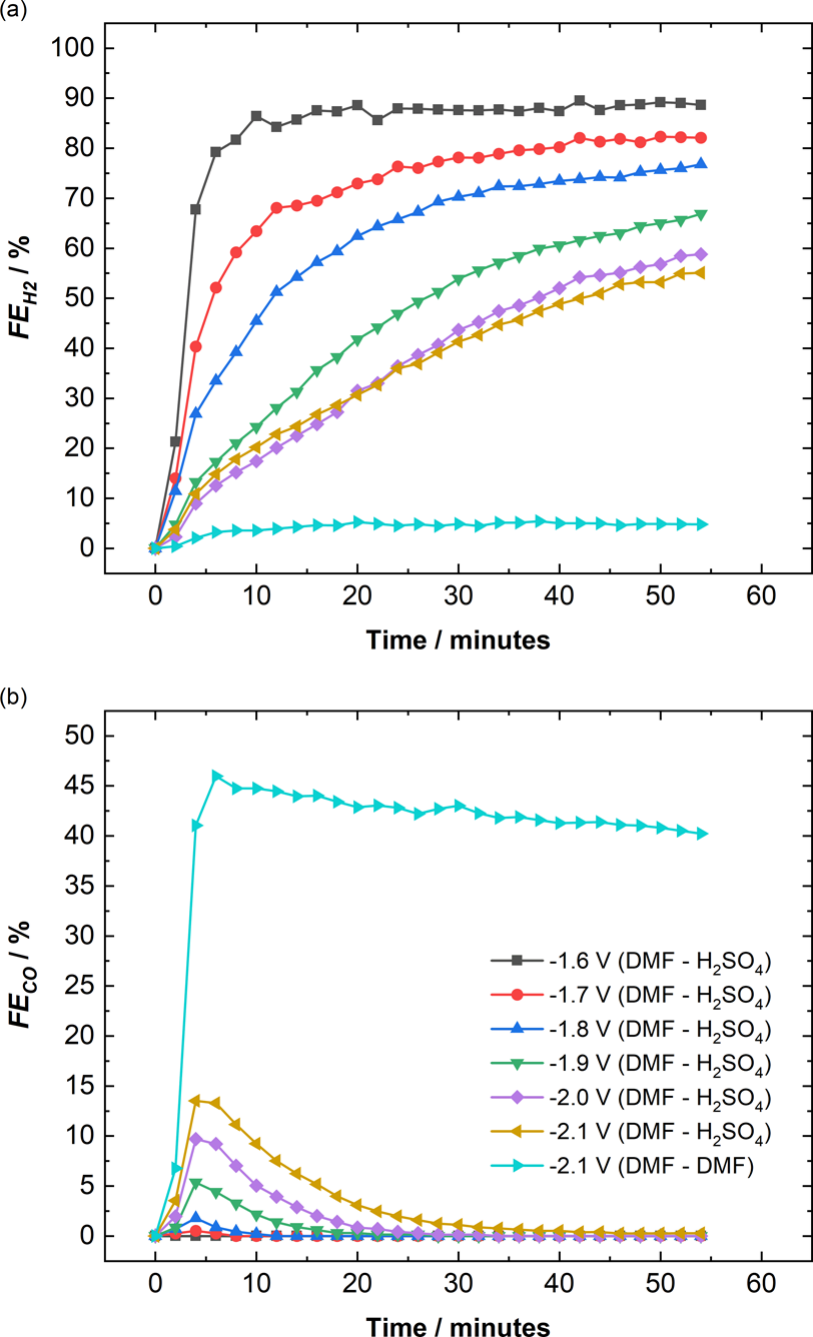
Faradaic efficiencies of the gaseous products (a) H_2_ and (b) CO, obtained over a potential range of −1.6 to −2.1 V during 1 h chronoamperometry experiments with 0.1 M TBAPF_6_ in pure DMF as a catholyte, 0.1 M H_2_SO_4_ as an anolyte, and a Nafion 117 membrane. The case with 0.1 M TBAPF_6_ in pure DMF as both catholyte and anolyte at −2.1 V is also shown.

To verify whether the first (less negative potential) reduction peaks observed in the CV measurements (−1.5 V for DMF) are related to CO_2_ reduction, chronoamperometry experiments are performed over a potential range of −1.6 to −1.8 V with 0.1 M TBAPF_6_ in pure DMF as the catholyte and 0.1 M H_2_SO_4_ as the anolyte to stay above the electrolyte breakdown potential. The FEs of all the gaseous products obtained during the 1 h experiments are shown in Figure 2 and Figure S3. The FE toward H_2_ is observed to be 89% at −1.6 V, and no other gaseous products are formed at this potential. At −1.7 V, the FE toward H_2_ decreased to 82% and a slight formation of CO, peaking at 1% FE is observed during the first 4 min of the experiment. The CO production is completely suppressed after 8 min, while H_2_ evolution steadily increased and reached a saturation after this point. A similar trend is again observed at −1.8 V, where the FE toward H_2_ further decreases to 75%, while the FE toward CO reaches a maximum of 2% after 4 min and is completely suppressed after 12 min.

To get further insights into the trend observed with these three potentials, experiments are performed at more negative potentials of −1.9 to −2.1 V with the same cell configuration. The FEs of all the gaseous products obtained at these potentials during the 1 h chronoamperometry experiments are also shown in Figure 2 and Figure S3. As the potential is increased to −1.9, −2.0, and −2.1 V, the trend continues and the FE toward H_2_ decreases to 64%, 56%, and 53%, while the peak FE of CO after 4 min increases to 5%, 10%, and 14%, respectively. It can also be seen that CO is detected for longer periods with increasing potential, i.e., 28, 34, and 58 min with −1.9, −2.0, and −2.1 V, respectively, before it is completely suppressed. Apart from H_2_ and CO, slight amounts of CH_4_ (FE = 0.25%) and C_2_H_4_ (FE = 0.7%) are formed at −2.1 V with their FEs peaking at the fourth minute (see Figure S3), similar to the trend observed with CO. Despite the fact that the FE toward H_2_ reduced with increasing potential, H_2_ production steadily increased over the duration of the experiments at all studied potentials (see Figure 2a). It is evident from the experiments that the H_2_ evolution eventually outcompetes CO_2_ reduction and dominates in all these cases. During the first minutes of the experiments, the proton availability near the surface of the Cu electrode is limited and the HER is effectively suppressed. However, due to the continuous migration of protons or diffusion of water from the anolyte to the catholyte though the membrane, the proton availability near the surface of the Cu electrode increases over the course of the experiments and the local environment favors H_2_ reduction over the CO_2_ reduction.^53−55^

To confirm the source of HER and to further analyze the effect of anolyte choice on the electrochemical CO_2_ reduction in non-aqueous electrolytes, the electrochemical experiment at −2.1 V is repeated with the same cell configuration, but by changing the anolyte from 0.1 M H_2_SO_4_ solution to 0.1 M TBAPF_6_ in DMF, similar to the catholyte. The FEs of all the gaseous products obtained during this 1 h CO_2_RR experiment are compared with the previous experiments in Figure 2 and Figure S3. The current densities obtained during all the above experiments are shown in Figure S4. The FE toward H_2_ decreased from 53% to 5%, while the peak FE of CO increased from 14% to 45% in the first 4–6 min, just by changing the anolyte to the non-aqueous electrolyte. The FE toward CO is stable, with only a slight drop of 5% during 1 h of electrolysis. In the case of CH_4_, the FE steadily increased and reached a peak value of 0.6% at the end of 1 h, while the FE toward C_2_H_4_ increased to 1.4% and remained stable over the duration of the experiment. This confirms that the preferential and dominating HER observed in the previous experiment originates from the continuous migration of protons or diffusion of water from the acidic anolyte to the catholyte via the cation exchange membrane (CEM), ultimately suppressing the CO_2_ reduction. Hence, the second cell configuration, with the same non-aqueous electrolyte as the catholyte and the anolyte, is used for all further experiments with DMF, NMP, and ACN.

### 3.3 Electrocatalytic Performance in Non-aqueous Electrolytes

**Figure 3:**
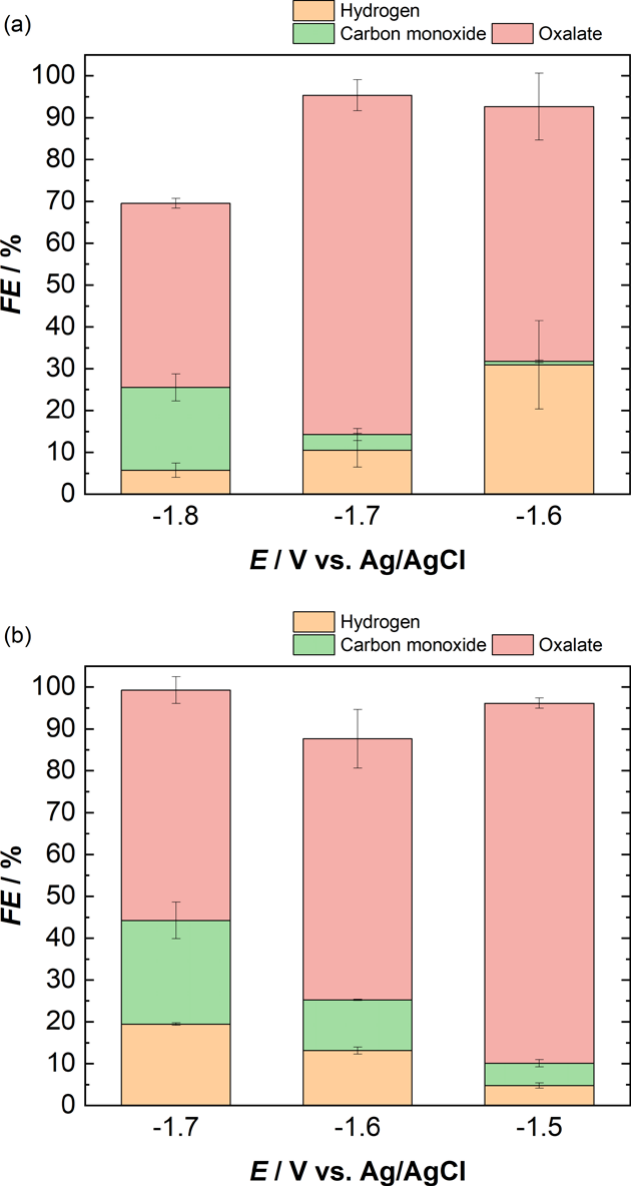
Faradaic efficiencies of all gaseous and liquid products obtained during chronoamperometry experiments over a potential range of (a) −1.6 to −1.8 V with 0.1 M TBAPF_6_ in DMF and (b) −1.5 to −1.7 V with 0.1 M TBAPF_6_ in NMP. The catholyte is the same as the anolyte in all experiments, and a Nafion 117 CEM membrane is used to separate the two compartments.

The electrocatalytic performance is determined by running chronoamperometry experiments over a potential range of −1.6 to −1.8 V with 0.1 M TBAPF_6_ in DMF as the catholyte as well as the anolyte. Similar experiments are also performed with NMP over a potential range of −1.5 to −1.7 V to stay above the electrolyte breakdown potential of NMP. The FEs of all the gaseous and liquid products obtained at these potentials using DMF and NMP are shown in Figure 3. Additionally, the current densities obtained are shown in Figure S5.

For DMF, the FE toward CO increases from 1% to 20% as the applied potential is increased from −1.6 to −1.8 V (see Figure 3a). The FE toward H_2_ follows an opposite trend and decreases from 31% to 6% in the same potential range. The major product observed over the potential range of −1.6 to −1.8 V is oxalate, with the FE toward oxalate peaking at 81% at −1.7 V. For NMP (see Figure 3b), the FE toward CO increases from 5% to 25% as the potential is increased from −1.5 to −1.7 V. However, the FE toward H_2_ also increases from 5% to 20% with the same potential increase, unlike with DMF. Oxalate is the major product over this potential range, similar to the DMF electrolyte, with the FE toward oxalate peaking at 86% at −1.5 V and then decreasing to 55% at −1.7 V.

In general, the CO_2_RR mechanism in organic electrolytes follows three different pathways, as shown below:^15,19,56^
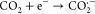
1
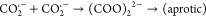
2
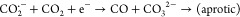
3
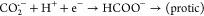
4where eq 1 represents the formation of a CO_2_^·–^ radical, eq 2 involves the dimerization of two CO_2_^·–^ radicals to form oxalate, and the pathway shown by eq 3 involves the disproportionation of a CO_2_^·–^ radical and a CO_2_ molecule to form CO and CO_3_^2–^. Both pathways in eqs 2 and 3 occur mainly in an aprotic environment. However, in the presence of water or protons, the pathway shown by eq 4 is followed, whereby the CO_2_^·–^ radical gets protonated to form formate. In the case of the pure DMF and NMP electrolytes, it is evident that the aprotic pathways are favored due to the limited availability of protons in the system.

**Figure 4:**
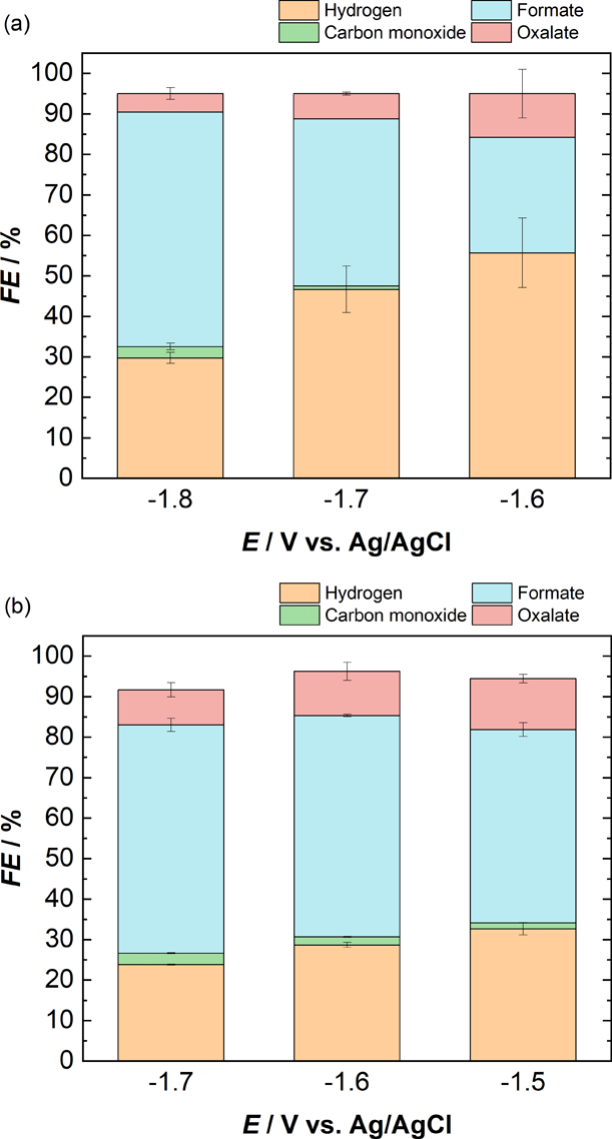
Faradaic efficiencies of all gaseous and liquid products obtained during chronoamperometry experiments over a potential range of (a) −1.6 to −1.8 V with 0.1 M TBAPF_6_ in 95% (v/v) DMF and (b) −1.5 V to −1.7 V with 0.1 M TBAPF_6_ in 95% (v/v) NMP. The catholyte is the same as the anolyte in all experiments, and a Nafion 117 CEM membrane is used to separate the two compartments.

To study the effect of water in these non-aqueous amide electrolytes, chronoamperometry experiments are performed with 0.1 M TBAPF_6_ in 95% (v/v) DMF and 95% (v/v) NMP with 5% (v/v) water over the same potential ranges (see Figure 4 for the FE and Figure S6 for the current densities). Figure 4a shows that the FE toward H_2_ increases significantly from 31% to 56% at −1.6 V in the case of 95% (v/v) DMF, compared to pure DMF. The FE toward oxalate decreases considerably from 81% to 6% at −1.7 V and the FE toward CO also decreases from 20% to 3% at −1.8 V, with the addition of 5% (v/v) water to the system. Formate is observed as the dominant CO_2_RR product, with the FE toward formate increasing from 28% to 58% as the applied potential is increased from −1.6 to −1.8 V. This is a strong indication that the protic pathway (eq 3) gains dominance over the aprotic pathways (eqs 1 and 2) in the presence of water. The effect of 5% (v/v) water in NMP can be seen in Figure 4b, where the trends observed for the production of H_2_, CO, oxalate, and formate are similar to those seen in 95% (v/v) DMF. However, formate is the major product obtained at all potentials in the case of 95% (v/v) NMP, with the FE toward formate increasing from 48% to 56% as the potential is increased from −1.5 to −1.7 V. The average current densities obtained with 5% (v/v) water addition almost doubled from −1.5 to −3.2 mA cm^–2^ and −0.7 to −1.5 mA cm^–2^ in the cases of DMF and NMP, respectively, especially at more negative potentials (see Figures S5 and S6). The increased current density is mainly due to the increased HER and formate production occurring in the presence of protons and water (see Figures S7 and S8).

**Figure 5:**
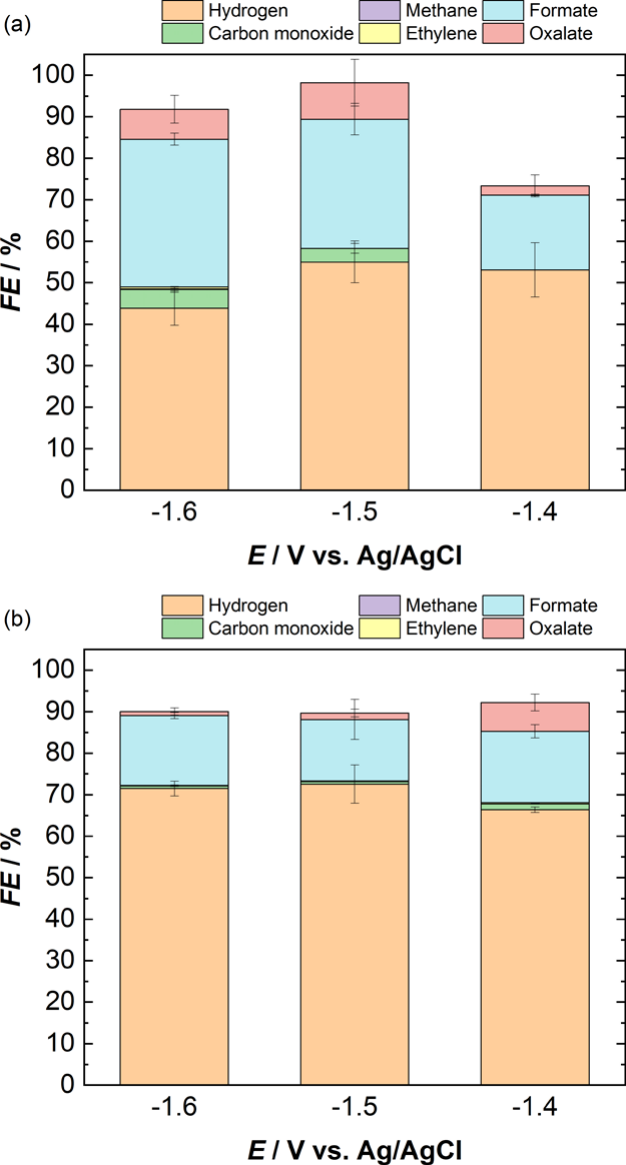
Faradaic efficiencies of all gaseous and liquid products obtained during chronoamperometry experiments over a potential range of −1.4 to −1.5 V with 0.1 M TBAPF_6_ in (a) ACN and (b) 95% (v/v) ACN. The catholyte is the same as the anolyte in all experiments, and a Nafion 117 CEM membrane is used to separate the two compartments.

**Figure 6:**
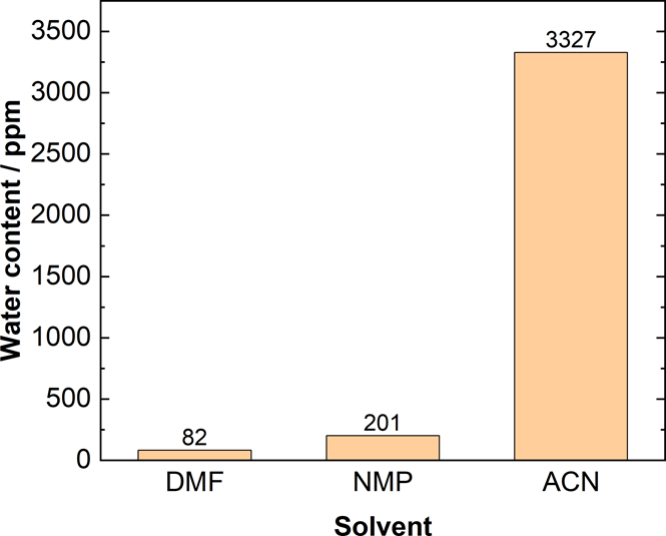
Water content present in the pure DMF, NMP, and ACN electrolytes extracted directly from the bottles, determined by Karl Fischer coulometric titration.

Similar experiments are also performed with 0.1 M TBAPF_6_ in pure ACN and 95% (v/v) ACN over a potential range of −1.4 to −1.6 V, to stay above the electrolyte breakdown potential. The FEs of all the products and the current densities obtained are shown in Figure 5 and Figure S9, respectively. For pure ACN, the major product obtained is H_2_, with the FE toward H_2_ peaking at 55% at −1.5 V and decreasing at more negative applied potentials. Formate is the major CO_2_RR product obtained with an FE increasing from 18% to 36% as the potential is increased from −1.4 to −1.6 V. The production of H_2_ and formate as major products in pure ACN implies the presence of a sufficient amount of protons in the system, so that the HER and also the protic pathway (eq 3) of CO_2_ reduction are favored. To verify the water content present in the pure non-aqueous electrolytes extracted from the bottle, Karl Fischer coulometric titration is performed. Figure 6 shows the amount of water present in the three non-aqueous electrolytes used in this study.

The water content present in the pure ACN electrolyte (3327 ppm) is almost 40 times higher than that in the pure DMF electrolyte (82 ppm), ultimately acting as a good proton source for the HER and also favoring the production of formate via the protic pathway. Moreover, the hygroscopic nature of ACN makes it difficult to remove the water completely from the system. The residual water present in the system could thus majorly contribute to the HER (see Figure S10).^18,51^ The presence of water is also reflected in the FE toward oxalate in pure ACN, which is <10%, while pure DMF and NMP produce oxalate with a FE > 80% at similar potentials. At −1.6 V, slight amounts of CH_4_ (FE = 0.15%) and C_2_H_4_ (FE = 0.45%) are produced alongside other CO_2_ reduction products. Since hydrocarbons are not typically expected during electrochemical CO_2_ reduction in non-aqueous electrolytes, there could be two possible ways in which these could be formed. The first possible pathway could be the aqueous CO_2_ reduction pathway, in the abundance of protons, where the CO_2_ gets adsorbed onto the Cu surface and undergoes multiple proton–electron transfers to produce various products including CH_4_ and C_2_H_4._^57,58^ The second possible mechanism could be a combination of the non-aqueous and the aqueous mechanism, where CO is initially produced via the disproportionation of a CO_2_^–^ radical and a CO_2_ molecule (eq 2) followed by the subsequent reduction of CO to various CO_2_ reduction products.

A similar product distribution can be seen in the case of 95% (v/v) ACN (see Figure 5b), but with an increase in hydrogen production and a decrease in CO_2_ reduction products. The FE toward H_2_ increased from 55% to 73% at −1.5 V, while the FE toward formate decreased from 36% to 17% at −1.6 V with the addition of 5% (v/v) water to the system. The FE toward oxalate and CO also decreased from 7% and 5%, respectively, to 1% at −1.6 V. The slight amount of hydrocarbons observed earlier with pure ACN at −1.6 V are no longer observed with 95% (v/v) ACN. However, trace amounts of CH_4_ (FE = 0.05%) and C_2_H_4_ (FE = 0.3%) can be observed at −1.4 V with 95% (v/v) ACN, which is not observed with pure ACN. This could be possibly due to the reduction of CO_2_ via the mechanism followed in the aqueous electrolytes, instead of the aprotic mechanism, with increased water content in the system. The average current densities obtained with ACN also increased considerably from −0.9 to −3.0 mA cm^–2^ with the addition of water, which can be attributed to the increased HER.

### 3.4 Comparison to Electrocatalytic Performance in an Aqueous Electrolyte

Chronoamperometry experiments are performed in a standard aqueous 0.1 M KHCO_3_ electrolyte over a potential range of −1.4 to −1.8 V to compare the results obtained with DMF, NMP, and ACN electrolytes with an aqueous electrolyte using the same experimental procedure.

**Figure 7:**
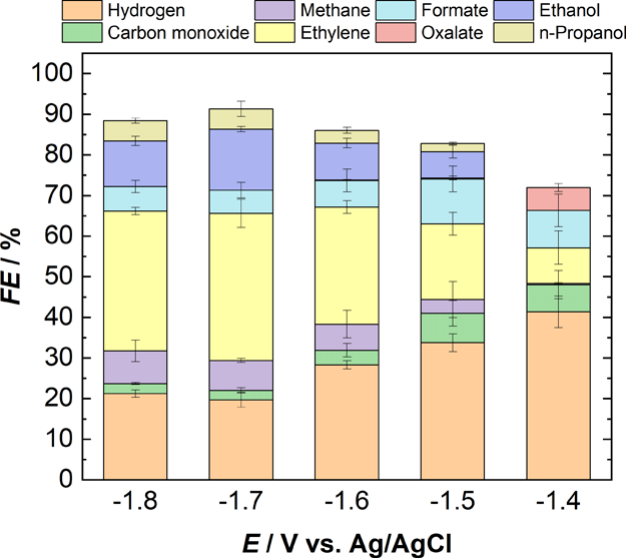
Faradaic efficiencies of major and minor gaseous and liquid products obtained during chronoamperometry experiments in standard aq. 0.1 M KHCO_3_ electrolyte over a potential range of −1.4 to −1.8 V. The catholyte is the same as the anolyte in all experiments, and a Selemion AEM membrane is used to separate the two compartments.

At −1.4 V, H_2_ is the major product with an FE of 41% (see Figure 7) while products such as CO, C_2_H_4_, and formate are detected only in smaller quantities (FE < 10%). At −1.7 V, the FEs toward C_2_H_4_ and ethanol reach a maximum of 36% and 15%, respectively, while the FE toward H_2_ reaches a minimum of 18%. Upon further increasing the potential to −1.8 V, the FE toward H_2_ starts to increase, while the FE toward C_2_H_4_ and ethanol starts to decrease. The production of CH_4_ is observed to increase with the potential, reaching an FE of 8% at −1.8 V. This trend is also in line with the results obtained in various other studies with 0.1 M KHCO_3_ solution on copper electrodes.^26,28,30,48^ Apart from these products, minor products (FE < 10%) such as CO, oxalate, and 1-propanol and trace products (FE < 1%) such as propane, isobutene, pentane, formate, glyoxal, and acetate are also detected over this potential range (see Figure S11). The average current density obtained over this potential range also increased from around −1 mA cm^–2^ at −1.4 V to around −5 mA cm^–2^ at −1.8 V (see Figure S12).

**Figure 8:**
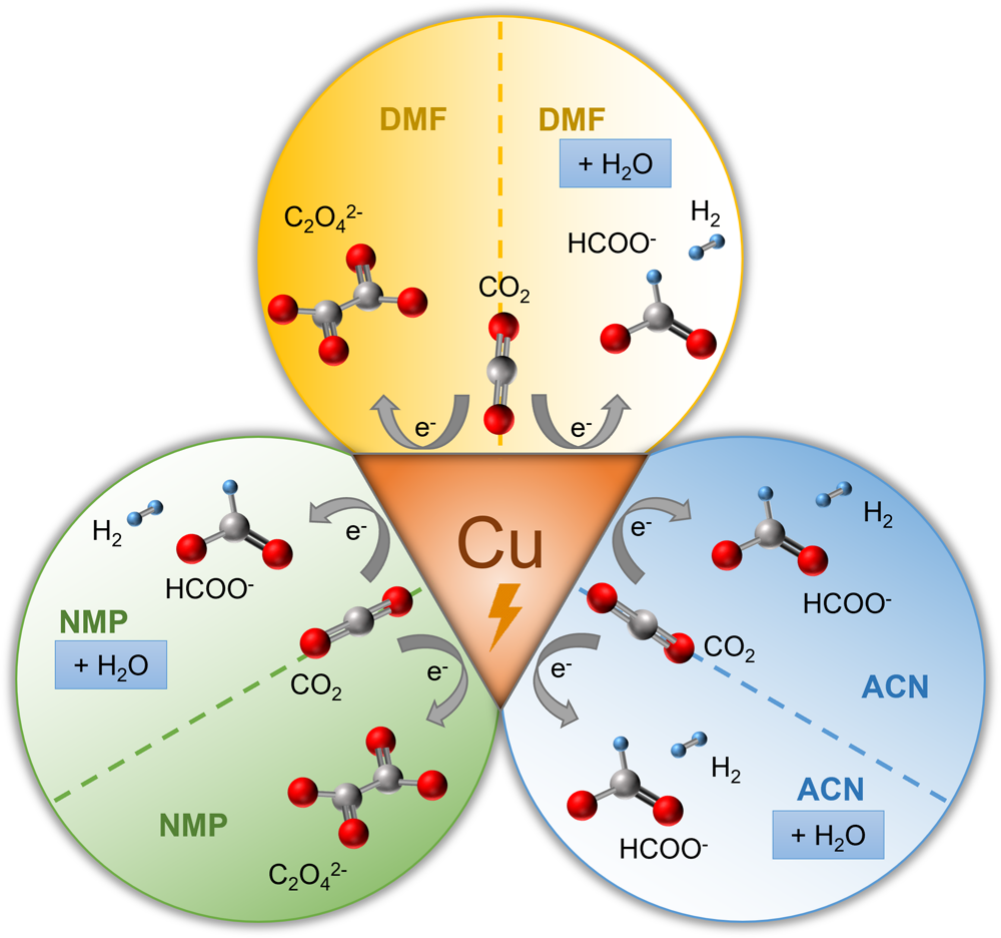
Schematic summarizing the major products produced on a copper electrode during electrochemical CO_2_ reduction with DMF, NMP, and ACN electrolytes with and without the addition of water.

Thus, for the production of hydrocarbons, especially C_2_H_4_, aqueous 0.1 KHCO_3_ proved to be more selective with the FE toward C_2_H_4_ reaching up to 36%, while pure amides such as DMF and NMP produced oxalate with a FE > 80% at potentials comparable to that required with the aqueous electrolytes. Both pure and 95% (v/v) ACN predominantly produced H_2_ and formate due to the residual and added water in the system, respectively. However, trace amounts (FE < 0.5%) of hydrocarbons are also observed with ACN over the studied potential range, as opposed to the case with DMF and NMP where no hydrocarbons are observed over the studied potential range. Initial experiments with DMF at potentials more negative than the electrolyte breakdown potential resulted in the production of trace amounts (FE < 1%) of hydrocarbons. However, a part of the electrons supplied at these potentials also goes into the reduction of the electrolyte, which is undesirable. It is evident from the results that at potentials less negative than the electrolyte breakdown potentials, CO_2_RR in pure amides follows the aprotic pathway and, in the presence of water or protons, follows the protic pathway, thus being predominantly selective to oxalate (see Figure 8). Our results show that organic electrolytes can aid in obtaining high selectivities toward oxalate and formate on a copper electrode, while aqueous electrolytes remain the optimal choice for the production of hydrocarbons and alcohols.

## 4 Conclusions

We have investigated the electrochemical CO_2_ reduction performance of copper in non-aqueous electrolytes based on DMF, NMP, and ACN. Experiments are performed with 0.1 M TBAPF_6_ in pure electrolytes and also with a 5% (v/v) water addition to determine its effect on the product distribution and selectivity toward hydrocarbons. Some of the challenges associated with the experimental setup in the case of non-aqueous electrolytes, such as the effect of aqueous/non-aqueous anolyte, membrane, and selection of potential window on the electrochemical CO_2_ reduction performance, are addressed in this study. Experiments with non-aqueous catholytes together with non-aqueous anolytes and a Nafion membrane effectively suppressed the HER and provided more stable results compared to experiments with non-aqueous catholytes and aqueous anolytes. Experiments with pure DMF and NMP mainly produced oxalate with FE > 80%; however, pure ACN mainly produced H_2_ and formate due to the presence of more residual water in the system. Addition of 5% (v/v) water to DMF and NMP resulted in increased HER and an increase in formate production. Meanwhile, in the case of ACN, HER just increased further while suppressing all other products with the addition of 5% (v/v) water. Experiments performed with standard aqueous 0.1 M KHCO_3_ to compare the product distribution over the same potential windows as that of the non-aqueous electrolytes show that the FE of C_2_H_4_ and ethanol reached a maximum of 36% and 15%, respectively, at −1.7 V (vs Ag/AgCl) and proved to be more selective toward the production of hydrocarbons. Therefore, we conclude that aqueous electrolytes remain a better choice for the production of hydrocarbons and alcohols on a copper electrode, while organic electrolytes based on DMF and NMP can be used to obtain high selectivity toward oxalate and formate.

## Supporting Information Available

Salt solubility table, onset potential table, schematic of experimental setup, faradaic efficiency and (partial) current density plots, and additional experimental details (PDF)
