# Supplementary material for: A Quantitative Analysis of Electrochemical CO2 Reduction on Copper in Organic Amide and Nitrile-Based Electrolytes
Source: J Phys Chem C Nanomater Interfaces. 2023 Jul 3;127(27):12857–66. doi: 10.1021/acs.jpcc.3c01955 (PMC10350962; doi:10.1021/acs.jpcc.3c01955)
Supplement: Supplementary file 1 — jp3c01955_si_001.pdf [file jp3c01955_si_001.pdf]

A Quantitative Analysis of Electrochemical CO_2 Reduction on Copper in Organic Amide and Nitrile-based Electrolytes

Asvin Sajeev Kumar[†], *Marilia Pupo*[†], *Kostadin V. Petrov*[‡], *Mahinder Ramdin*[†], *J. Ruud van Ommen*[‡], *Wiebren de Jong*[†], *Ruud Kortlever*[†*]

[†]Department of Process & Energy, Faculty of Mechanical, Maritime & Materials Engineering, Delft University of Technology, Leeghwaterstraat 39, 2628 CB Delft, The Netherlands

[‡]Department of Chemical Engineering, Faculty of Applied Sciences, Delft University of Technology, Van der Maasweg 9, 2629 HZ Delft, The Netherlands

*Email: R.Kortlever@tudelft.nl

Supporting Information

Table S1. Solubility of 0.1 M $KHCO_3$ and 0.1 M $TBAPF_6$ in various dilutions (v/v) of DMF, NMP and ACN electrolytes.

**Salt**	**Solvent**	**Solvent Concentration**				
		**20%**	**40%**	**60%**	**80%**	**100%**
**0.1 M $KHCO_3$**	**ACN**	soluble	soluble	partially	insoluble	insoluble
	**DMF**	soluble	partially	insoluble	insoluble	insoluble
	**NMP**	soluble	partially	insoluble	insoluble	insoluble
**0.1 M $TBAPF_6$**	**ACN**	insoluble	partially	soluble	soluble	soluble
	**DMF**	insoluble	insoluble	partially	soluble	soluble
	**NMP**	insoluble	insoluble	partially	soluble	soluble

Table S2. CO_2RR onset and electrolyte breakdown potentials observed from CVs for the pure and 95% (v/v) DMF, NMP and ACN electrolytes.

**Onset Potential**	**CO_2RR (V vs. Ag/AgCl)**	**Electrolyte Breakdown (V vs. Ag/AgCl)**
**DMF**	-1.4	-1.9
**95% DMF**	-1.5	-1.9
**NMP**	-1.3	-1.8
**95% NMP**	-1.2	-1.8
**ACN**	-1.1	-1.7
**95% ACN**	-1.2	-1.7

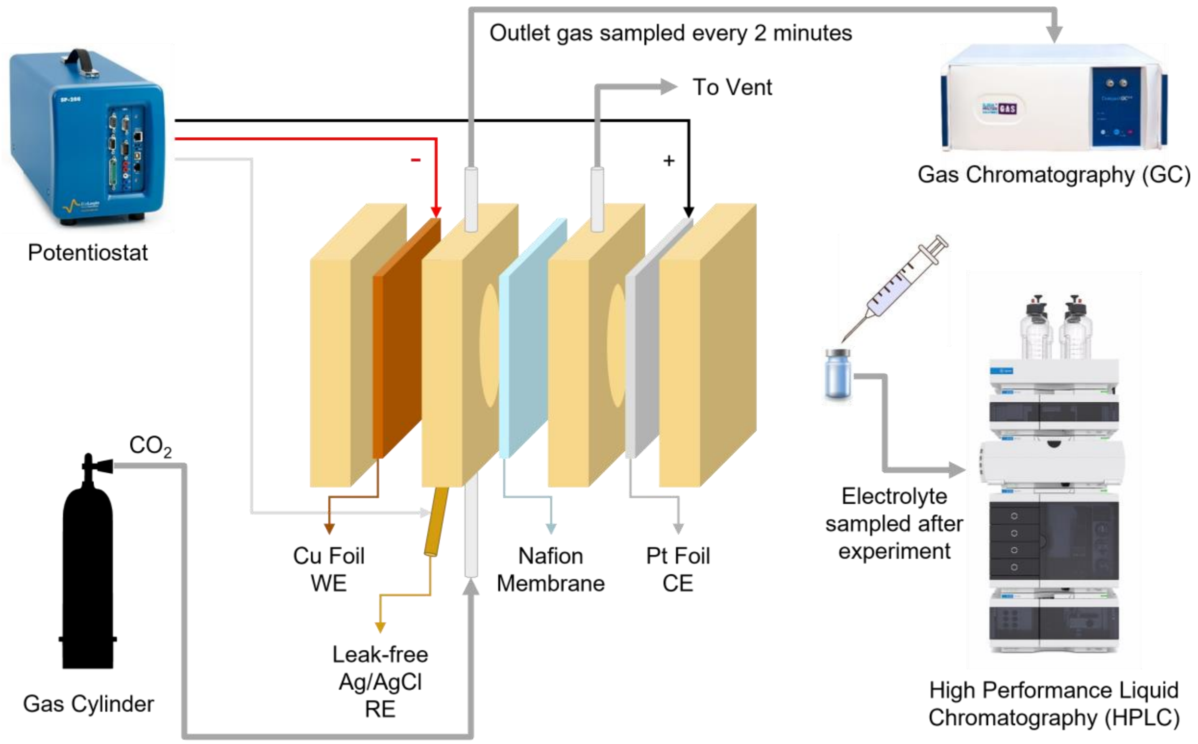

Figure S1. Schematic of the cell geometry and the experimental setup used for the chronoamperometry measurements. For cyclic voltammetry, the gas flow is stopped before the start of the measurements.

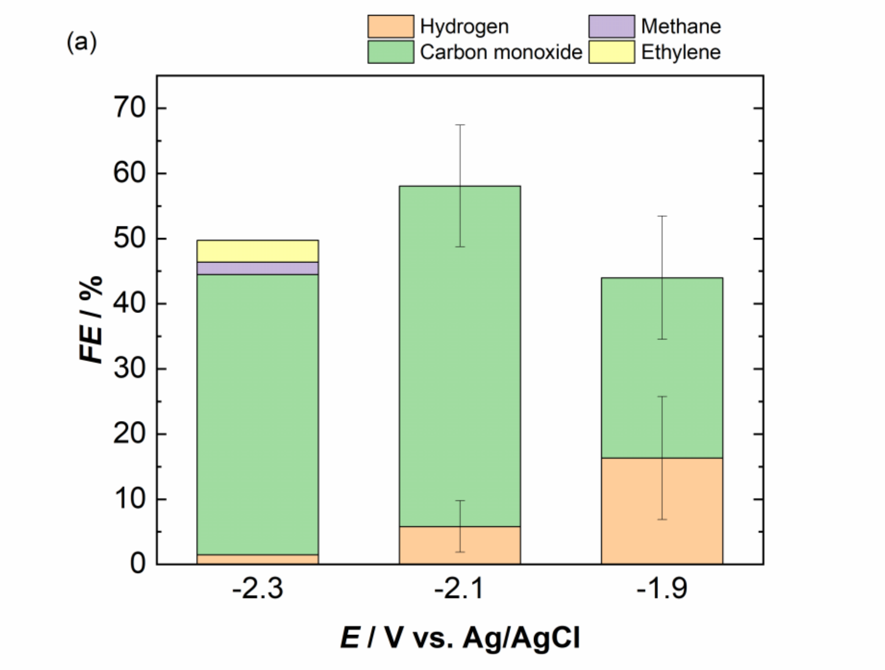

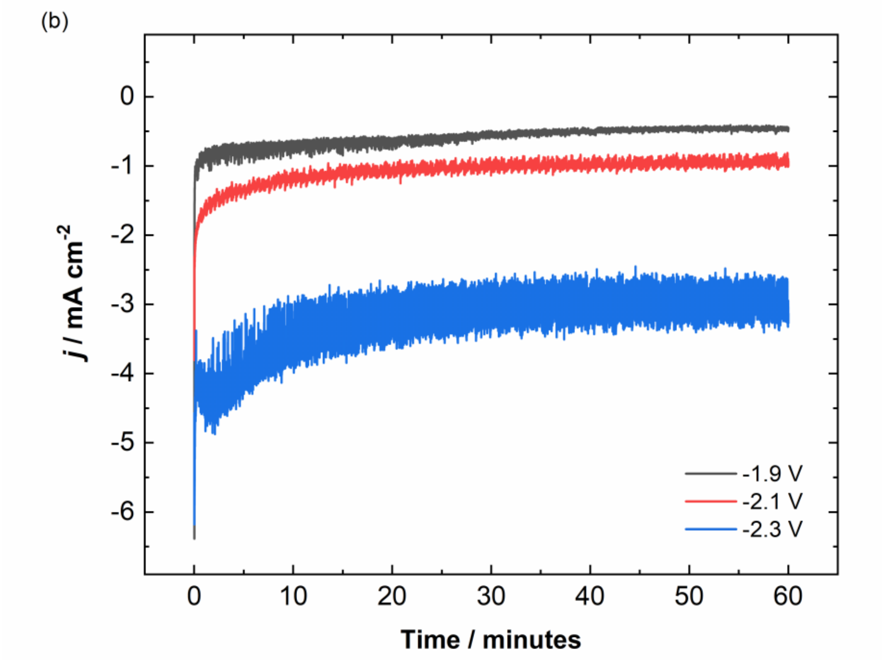

Figure S2. (a) Faradaic efficiencies of the gaseous products and (b) the current densities obtained during chronoamperometry experiments in pure DMF electrolyte at applied potentials of -1.9 V, -2.1 V and -2.3 V (vs. Ag/AgCl), using a Selemion anion exchange membrane to separate the compartments.

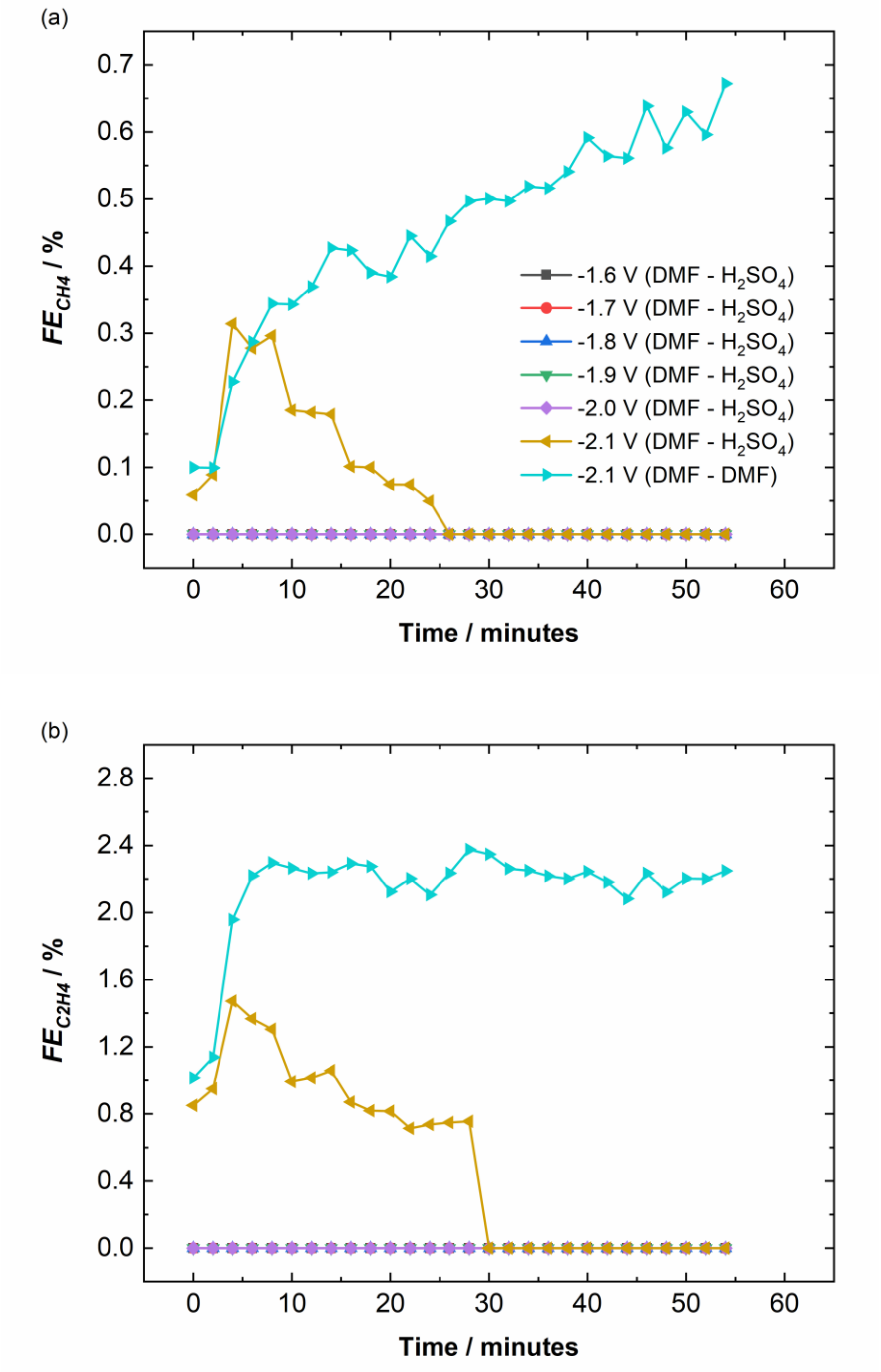

Figure S3. Faradaic efficiencies of the gaseous products (a) CH_4 and (b) C_2H_4, obtained over a potential range of -1.6 V to -2.1 V (vs. Ag/AgCl) during 1 hour chronoamperometry experiments with 0.1 M $TBAPF_6$ in pure DMF as catholyte, 0.1 M H_2SO_4 as anolyte and Nafion 117 membrane. The case with 0.1 M $TBAPF_6$ in pure DMF as both catholyte and anolyte at -2.1 V (vs. Ag/AgCl) is also shown.

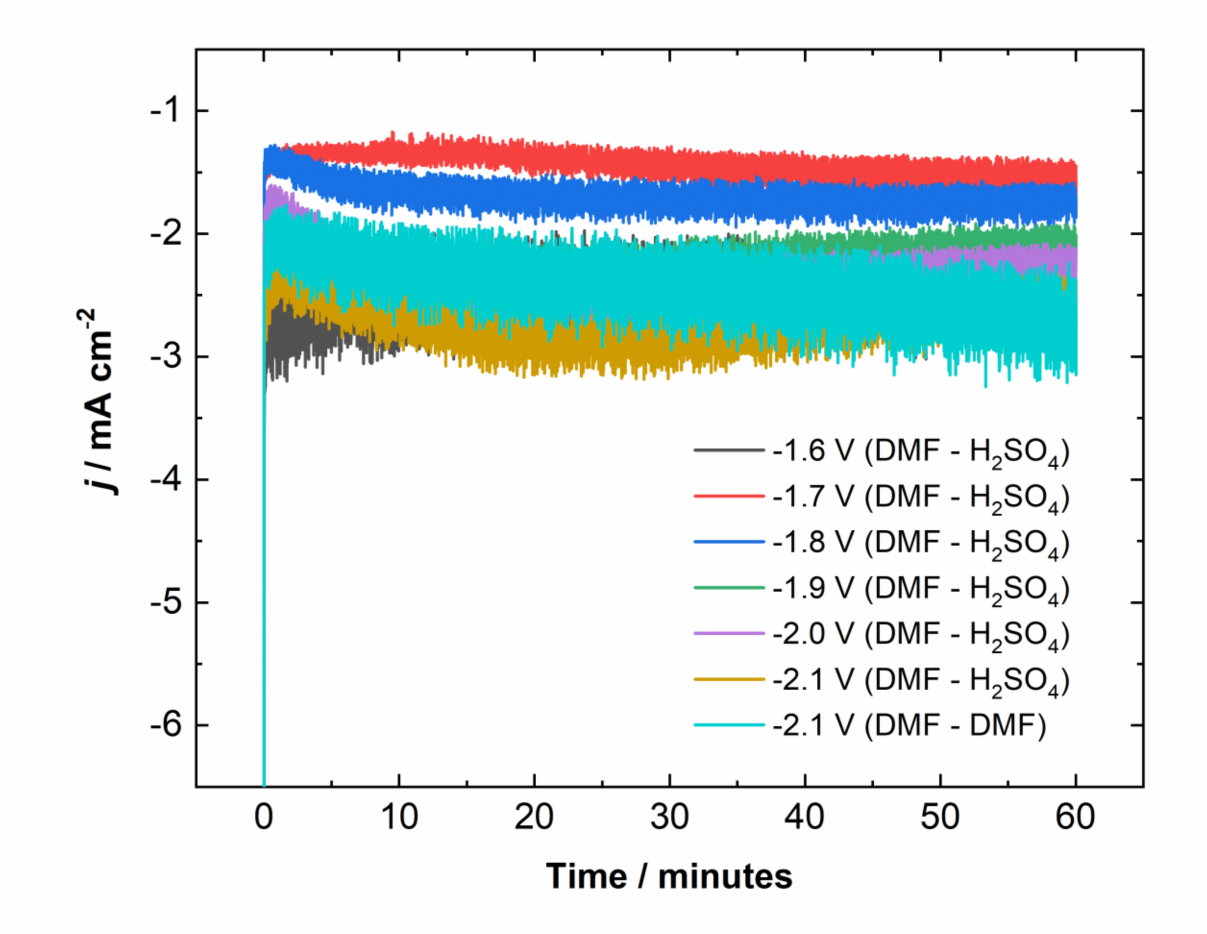

Figure S4. Current densities obtained over a potential range of -1.6 V to -2.1 V (vs. Ag/AgCl) during the 1 hour chronoamperometry experiments with 0.1 M $TBAPF_6$ in pure DMF as catholyte, 0.1 M H_2SO_4 as anolyte and Nafion 117 CEM membrane. The case with 0.1 M $TBAPF_6$ in pure DMF as both catholyte and anolyte at -2.1 V (vs. Ag/AgCl) is also shown.

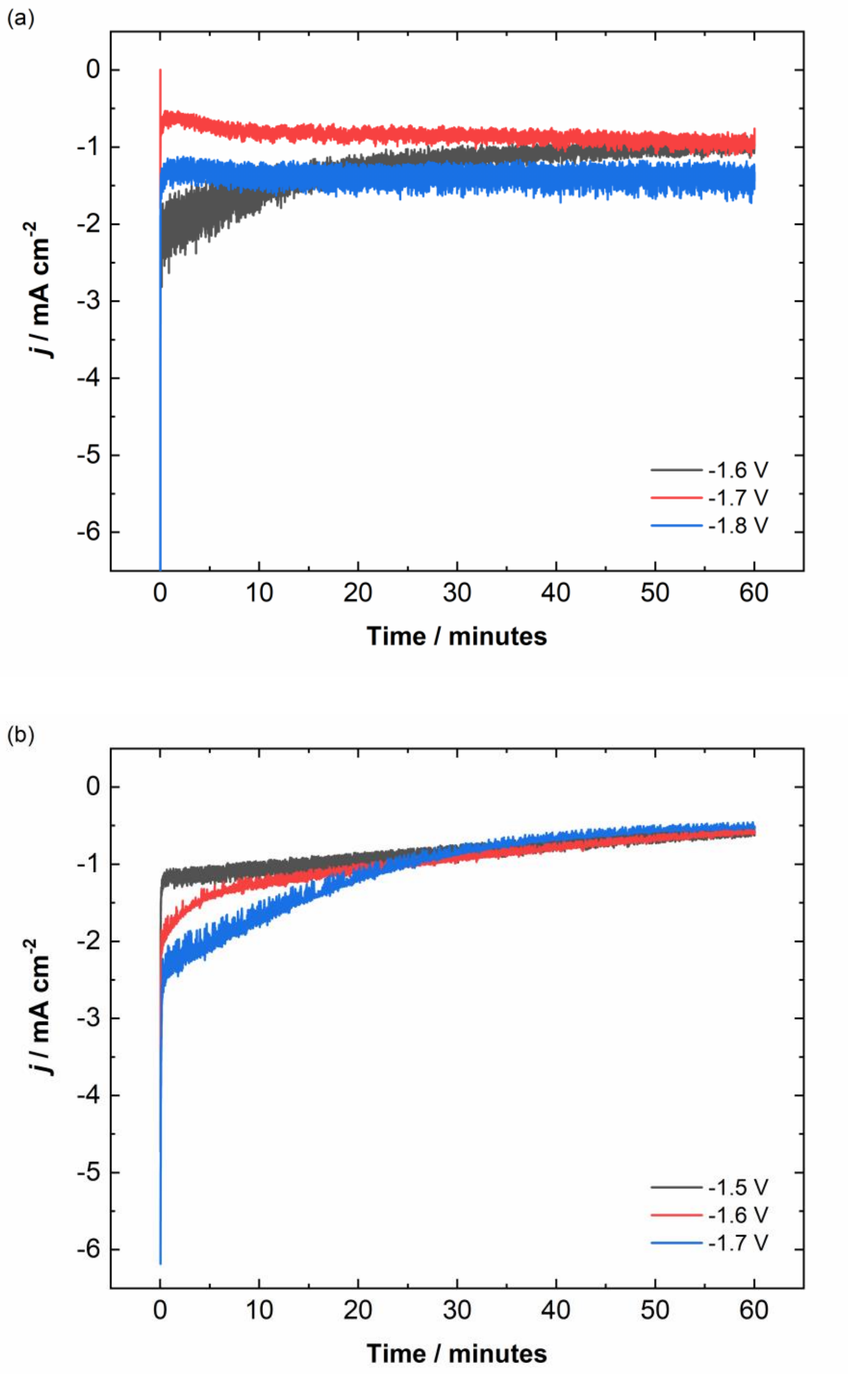

Figure S5. Current densities obtained during chronoamperometry experiments over a potential range of (a) -1.6 V to -1.8 V (vs. Ag/AgCl) with 0.1 M $TBAPF_6$ in pure DMF and (b) -1.5 V to -1.7 V (vs. Ag/AgCl) with 0.1 M $TBAPF_6$ in pure NMP. The catholyte is same as the anolyte in all experiments and Nafion 117 CEM membrane is used to separate the two compartments.

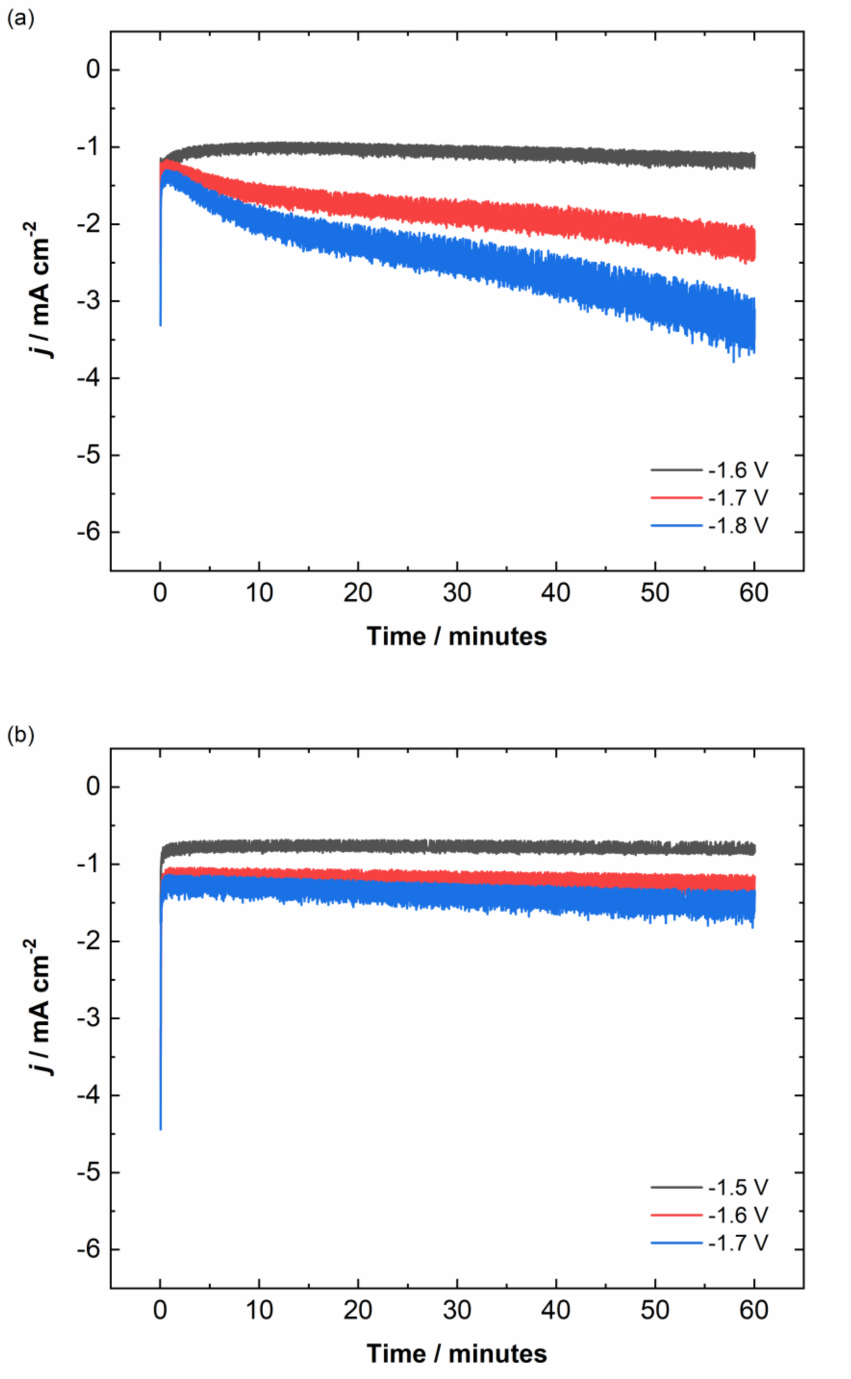

Figure S6. Current densities obtained during chronoamperometry experiments over a potential range of (a) -1.6 V to -1.8 V (vs. Ag/AgCl) with 0.1 M $TBAPF_6$ in 95% (v/v) DMF and (b) -1.5 V to -1.7 V (vs. Ag/AgCl) with 0.1 M $TBAPF_6$ in 95% (v/v) NMP. The catholyte is same as the anolyte in all experiments and Nafion 117 CEM membrane is used to separate the two compartments.

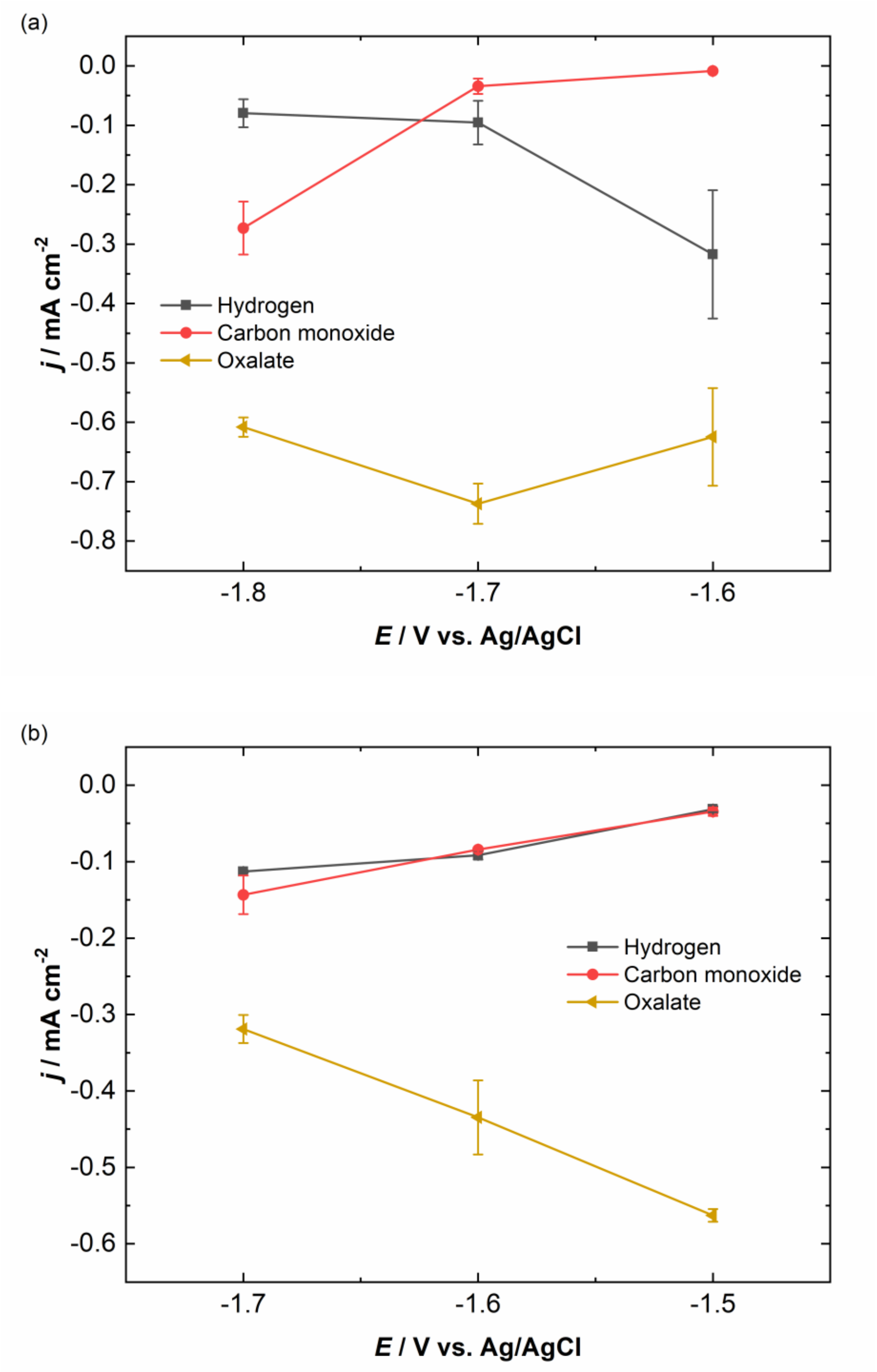

Figure S7. Partial current densities of all gaseous and liquid products obtained during chronoamperometry experiments over a potential range of (a) -1.6 V to -1.8 V (vs. Ag/AgCl) with 0.1 M $TBAPF_6$ in pure DMF and (b) -1.5 V to -1.7 V (vs. Ag/AgCl) with 0.1 M $TBAPF_6$ in pure NMP. The catholyte is same as the anolyte in all experiments and Nafion 117 CEM membrane is used to separate the two compartments.

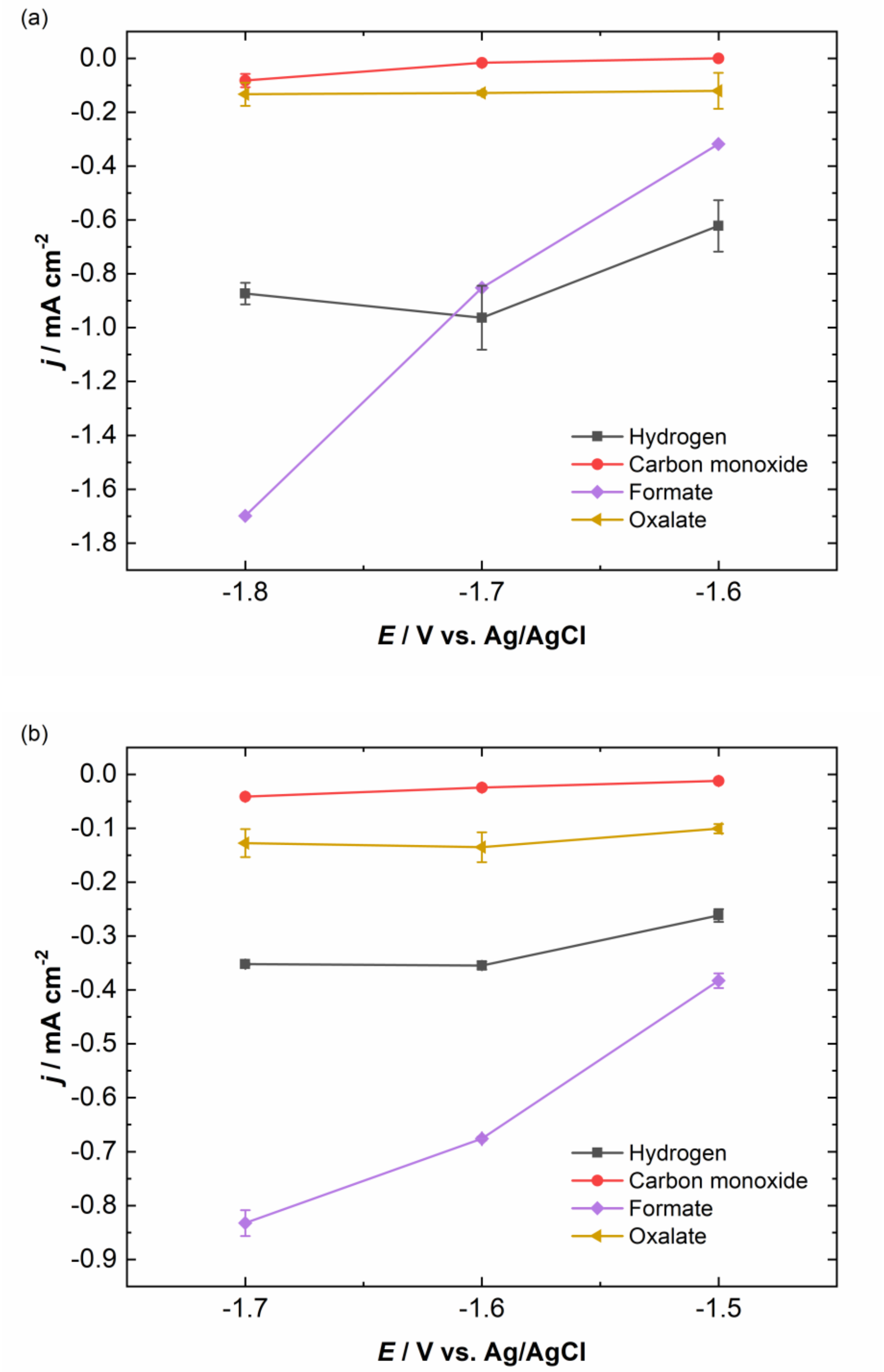

Figure S8. Partial current densities of all gaseous and liquid products obtained during chronoamperometry experiments over a potential range of (a) -1.6 V to -1.8 V (vs. Ag/AgCl) with 0.1 M $TBAPF_6$ in 95% (v/v) DMF and (b) -1.5 V to -1.7 V (vs. Ag/AgCl) with 0.1 M $TBAPF_6$ in 95% (v/v) NMP. The catholyte is same as the anolyte in all experiments and Nafion 117 CEM membrane is used to separate the two compartments.

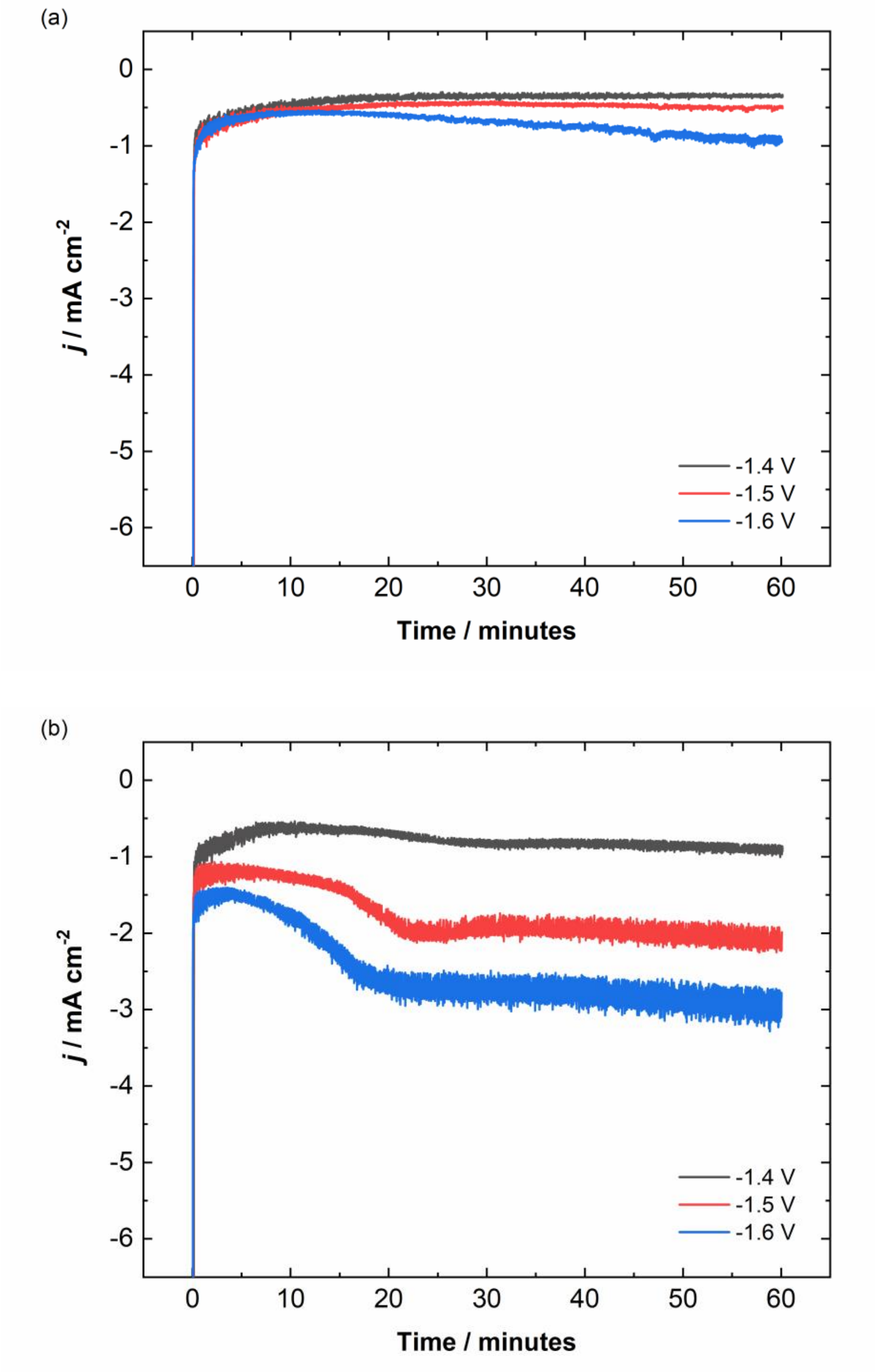

Figure S9. Current densities obtained during chronoamperometry experiments over a potential range of -1.4 V to -1.5 V (vs. Ag/AgCl) with 0.1 M $TBAPF_6$ in (a) pure ACN and (b) 95% (v/v) ACN. The catholyte is same as the anolyte in all experiments and Nafion 117 CEM membrane is used to separate the two compartments.

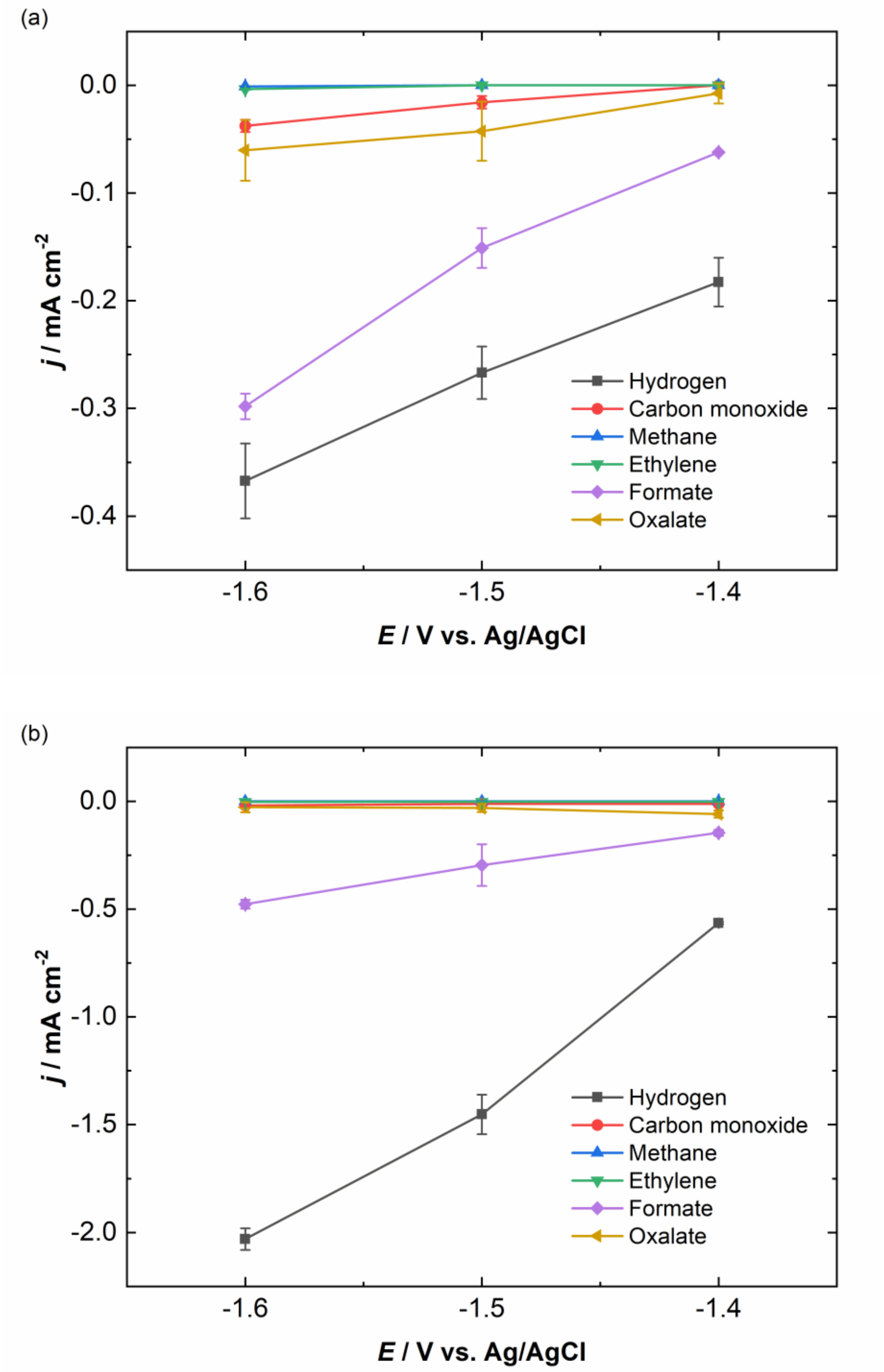

Figure S10. Partial current densities of all gaseous and liquid products obtained during chronoamperometry experiments over a potential range of -1.4 V to -1.5 V (vs. Ag/AgCl) with 0.1 M $TBAPF_6$ in (a) pure ACN and (b) 95% (v/v) ACN. The catholyte is same as the anolyte in all experiments and Nafion 117 CEM membrane is used to separate the two compartments.

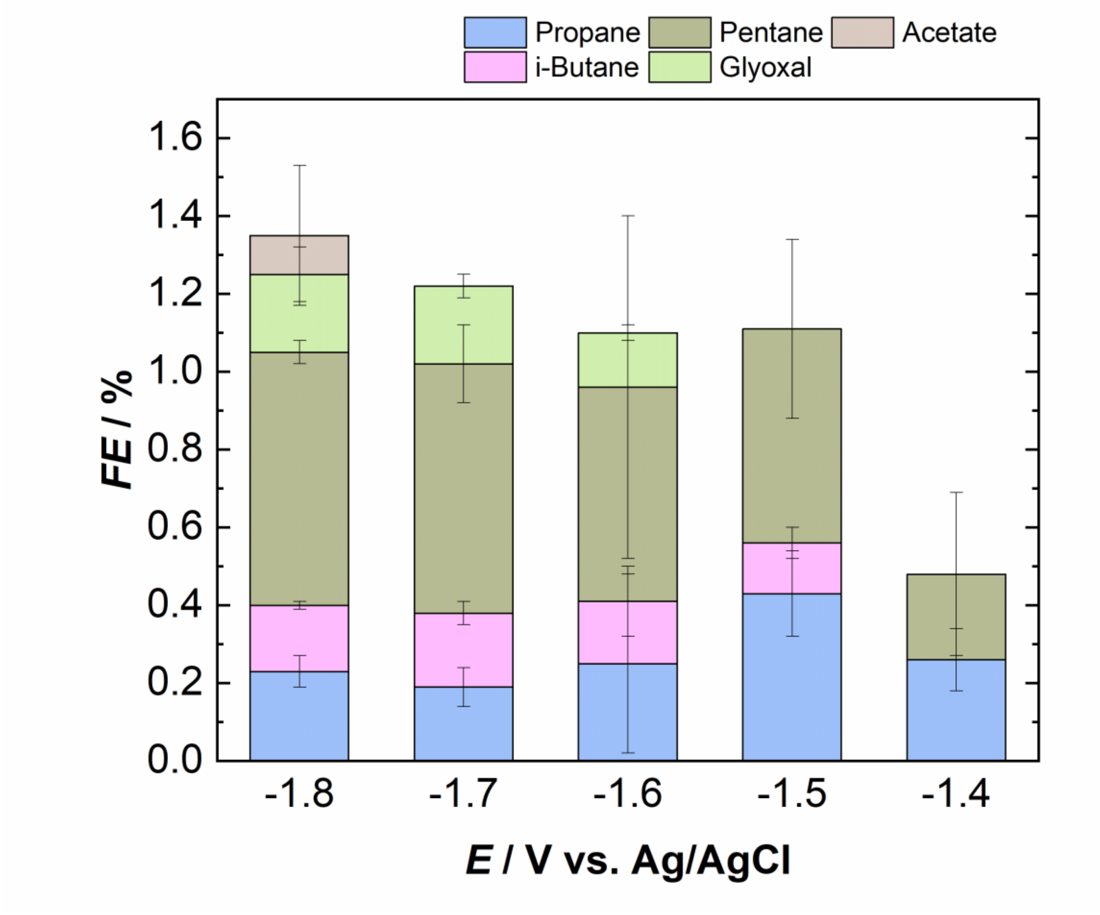

Figure S11. Faradaic efficiencies of trace gaseous and liquid products obtained during chronoamperometry experiments in standard aq. 0.1 M $KHCO_3$ over a potential range of -1.4 V to -1.8 V (vs. Ag/AgCl). The catholyte is same as the anolyte in all experiments and Selemion AEM membrane is used to separate the two compartments.

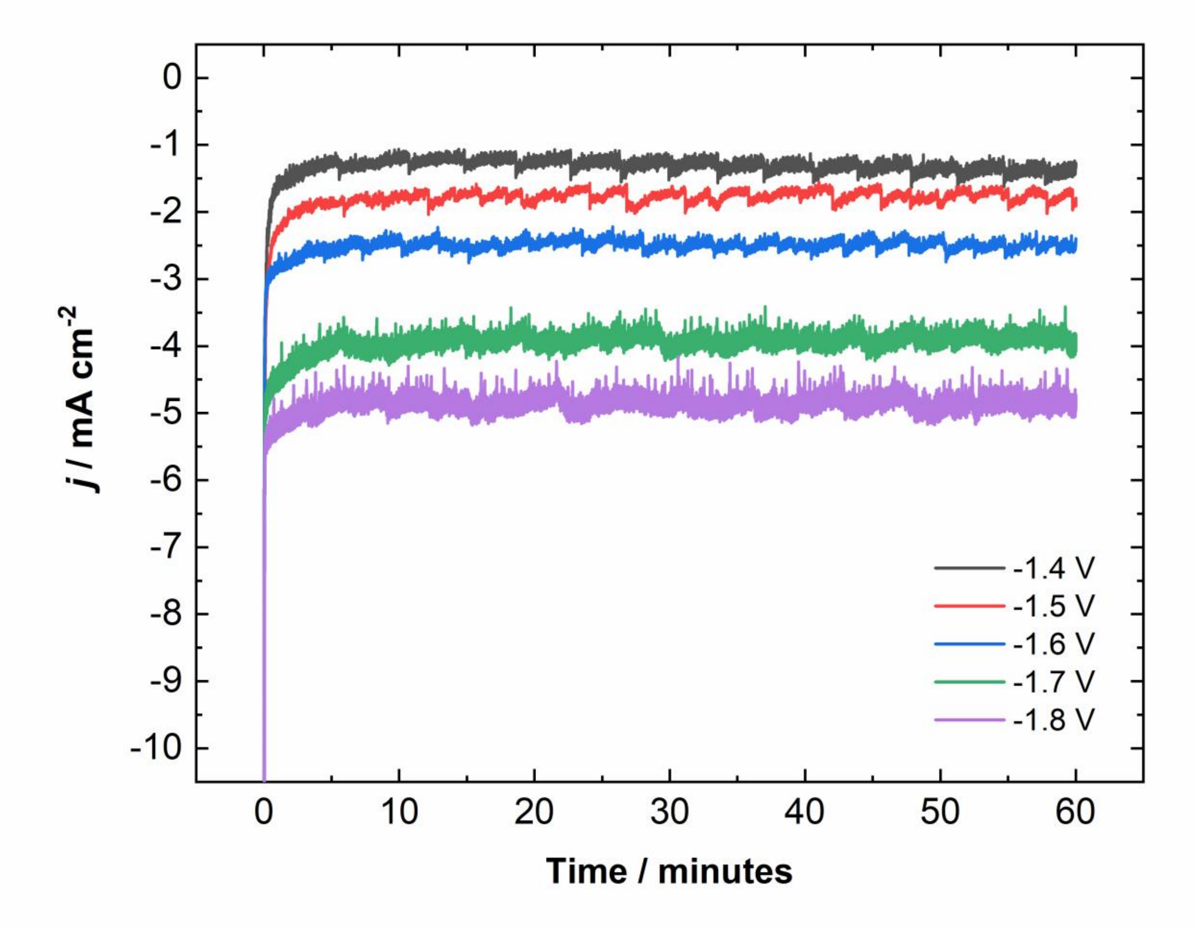

Figure S12. Current densities obtained during chronoamperometry experiments in standard aq. 0.1 M $KHCO_3$ over a potential range of -1.4 V to -1.8 V (vs. Ag/AgCl). The catholyte is same as the anolyte in all experiments and Selemion AEM membrane is used to separate the two compartments.

GC Calibration and Analysis

The component peaks in the GC chromatogram of the experimental sample are compared with the calibration peaks of the reference components to determine the type of component (by comparing the retention time) and its concentration (by comparing the area). The chromatogram obtained for one of the calibration gas mixtures with a composition of 100 ppm H_2, 1000 ppm CO, 3000 ppm CH_4, 8000 ppm C_2H_4 and rest CO_2 is shown in Figure S13. The retention times of CH_4 and C_2H_4 (FID_Ch1) are around 0.600 and 0.628 min, respectively, while the retention times of CO (TCD_Ch2) and H_2 (TCD_Ch3) are around 1.177 min and 0.521 min, respectively. Calibration curves are generated by plotting the concentrations of each of these components against their obtained areas with calibration gas mixtures of varying concentrations, such as 50 ppm, 100 ppm, 1000 ppm, 3000 ppm and 8000 ppm. The slopes of the calibration curves (Figure S14), m_{H2}, m_{CO}, m_{CH4}, and m_{C2H4}, are then used to determine the concentrations of each of these components in the experimental sample, using the formula:

$$C_x = A_x / m_x \qquad (x = H_2, CO, CH_4, C_2H_4)$$

where, C_x is the concentration in ppm, A_x is the area of the component peak in pA min (FID) or mV min (TCD) and m_x is the slope of the calibration curve for the component x. Calibration of C_{3+} gases are performed only with a single gas concentration and hence is less accurate compared to the basic gases mentioned above. However, the contribution of C_{3+} gases in the obtained product distribution is not significant and hence the error introduced is negligible.

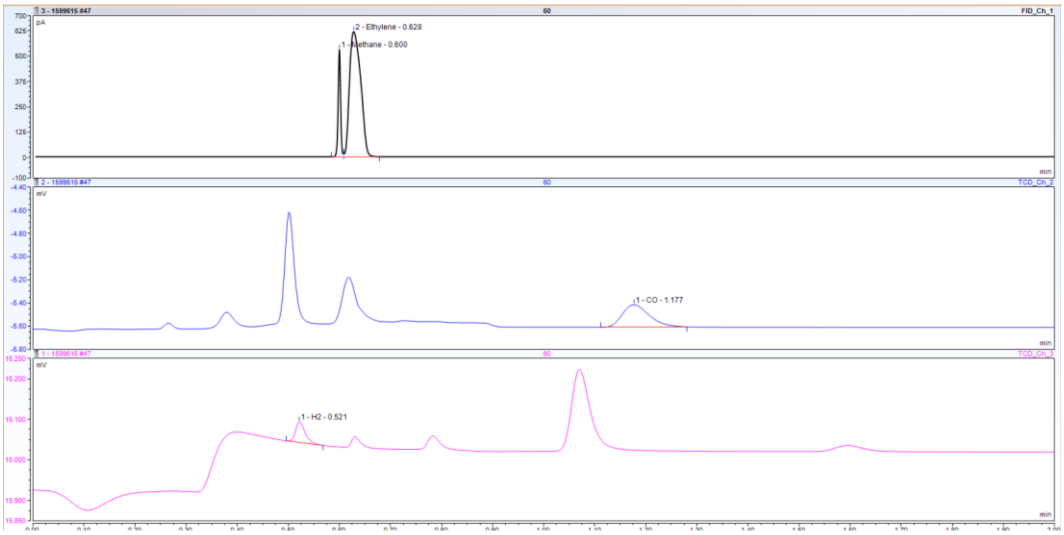

Figure S13. Chromatogram obtained for a calibration gas mixture using GC with a composition of 100 ppm H_2, 1000 ppm CO, 3000 ppm CH_4, 8000 ppm C_2H_4 and rest CO_2.

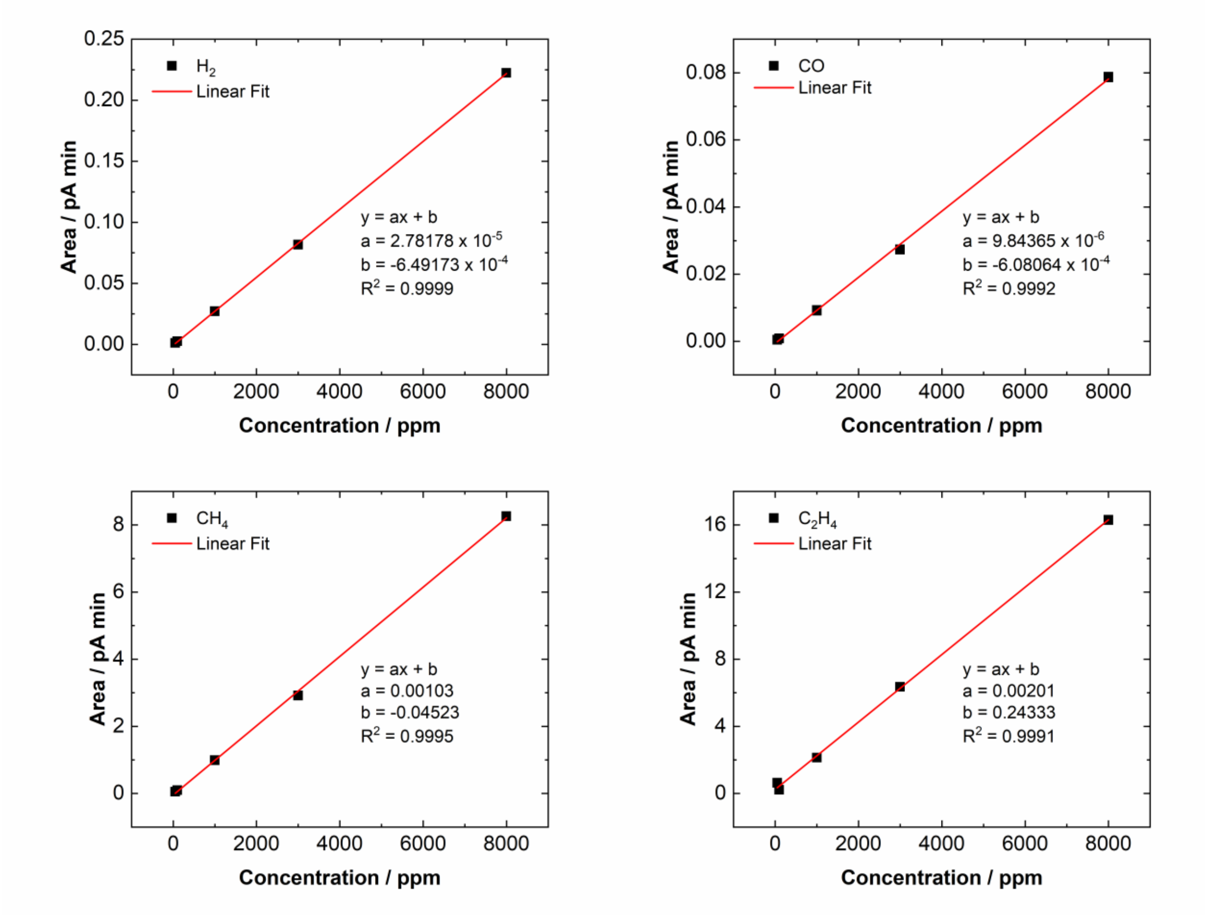

Figure S14. Calibration curves generated for H_2, CO, CH_4 and C_2H_4 using compact GC.

HPLC Calibration and Analysis

The product peaks in the HPLC chromatogram of the experimental sample are compared with the calibration peaks of the reference components to determine the type of component and its concentration. The chromatogram obtained for a sample containing ethanol is shown in Figure S15. The retention time of the ethanol peak is around 39-40 minutes. Calibration curves are generated by plotting the concentrations of each component against their obtained areas for various concentrations of the component, such as 0.1 mM, 0.5 mM, 1 mM, 5 mM, 10 mM, 25 mM and 50 mM. The slopes of the calibration curves (Figure S16, S17), m_y, are then used to determine the concentrations of each component in the experimental sample, using the formula:

$C_y = A_y / m_y$ (*y = Oxalate, glyoxal, formate, acetate, ethylene glycol, acetaldehyde, methanol, ethanol, acetone, propionaldehyde, 2-propanol, 1-propanol*)

where, C_y is the concentration of the product in mM and A_y is the area of the product peak.

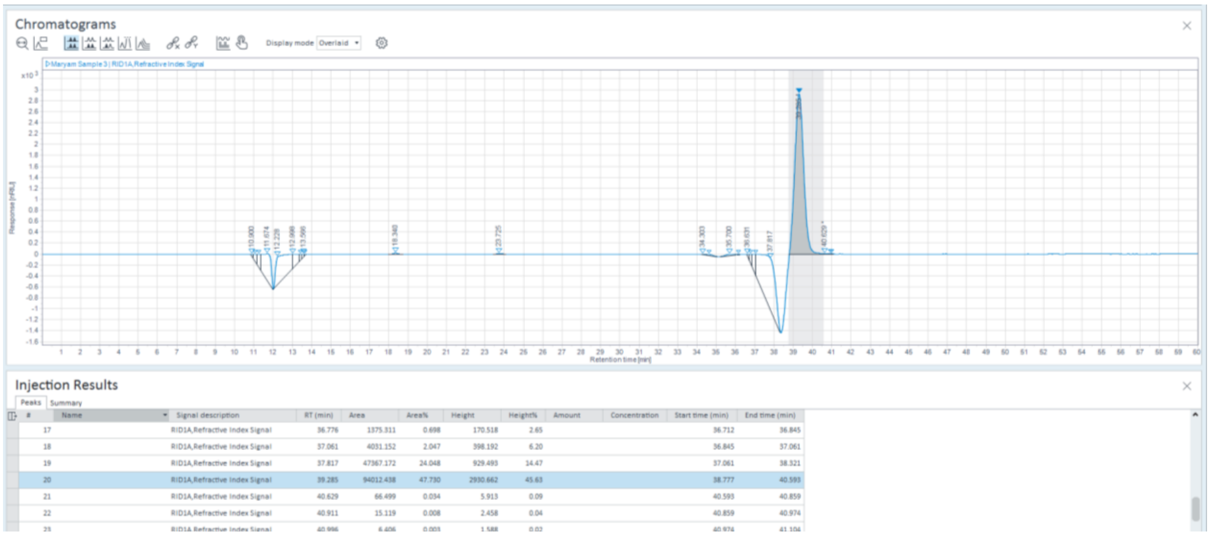

Figure S15. Chromatogram obtained using HPLC for a sample containing ethanol.

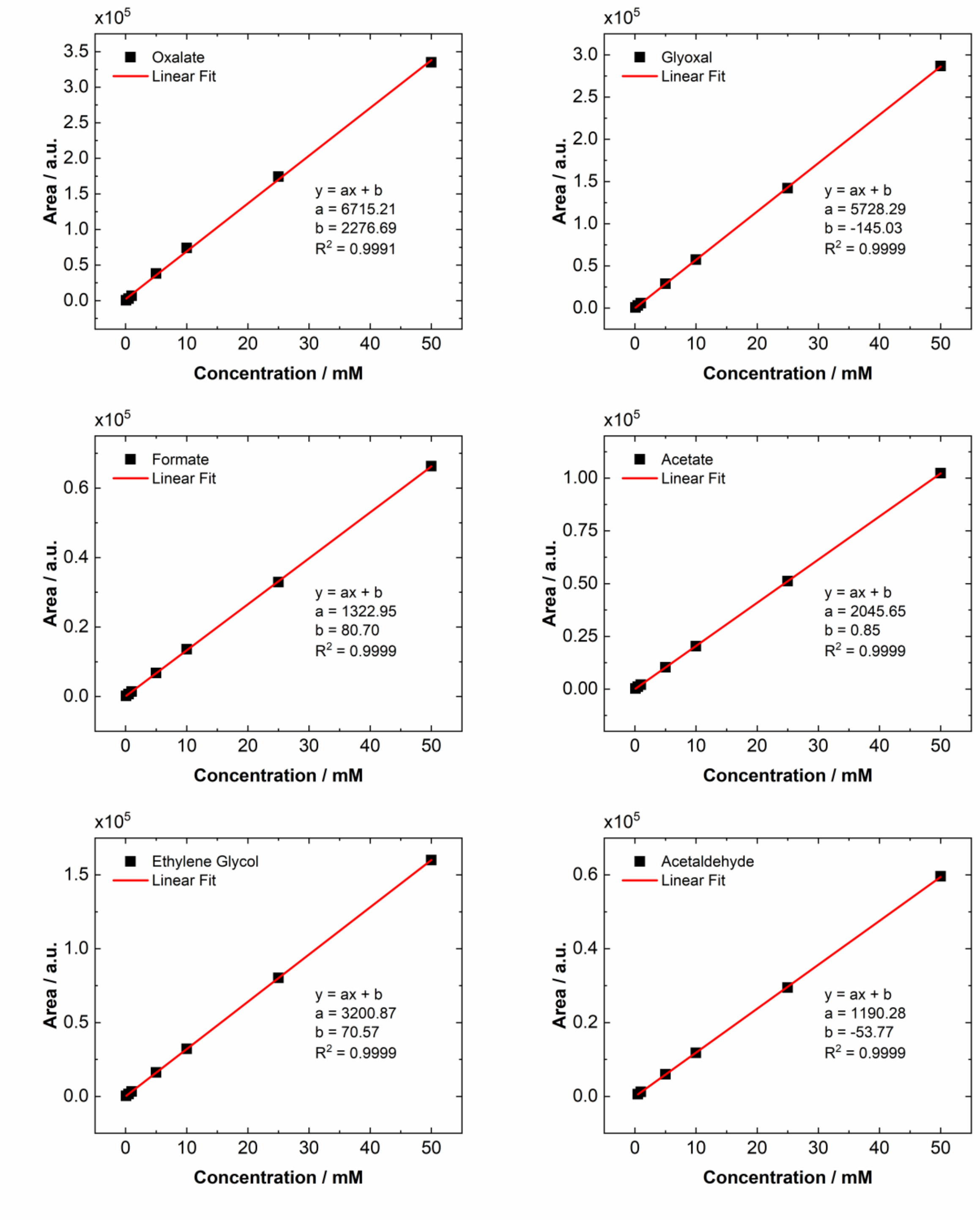

Figure S16. Calibration curves generated for oxalate, glyoxal, formate, acetate, ethylene glycol and acetaldehyde using HPLC.

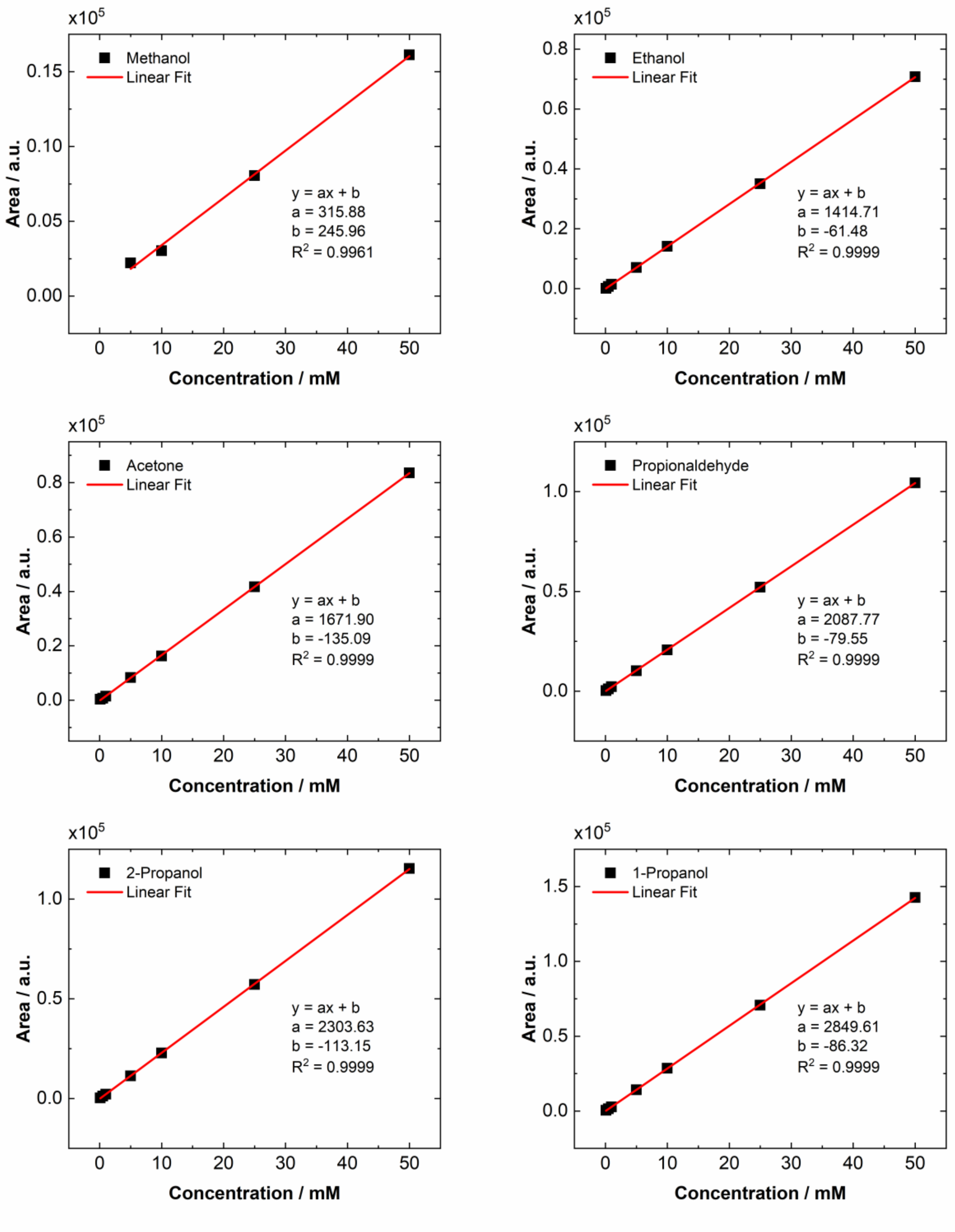

Figure S17. Calibration curves generated for methanol, ethanol, acetone, propionaldehyde, 2-propanol and 1-propanol using HPLC.

IC Calibration and Analysis

The calibration of the liquid products in IC is performed in the same way as the GC and the HPLC. Figure S18 shows the chromatogram obtained for various calibration samples. The retention time of formate is around 4.5 minutes and that of oxalate is around 16.8 minutes. Calibration curves are generated using 0.1 mM, 1.5 mM, 6 mM and 7.2 mM concentrations of the components. The slopes of the calibration curves (Figure S19) m_z, are then used to determine the concentrations of each component in the experimental sample, using the formula:

$$C_z = A_z / m_z \qquad (z = Formate,\ oxalate)$$

where, C_z is the concentration of the product in mM and A_z is the area of the product peak.

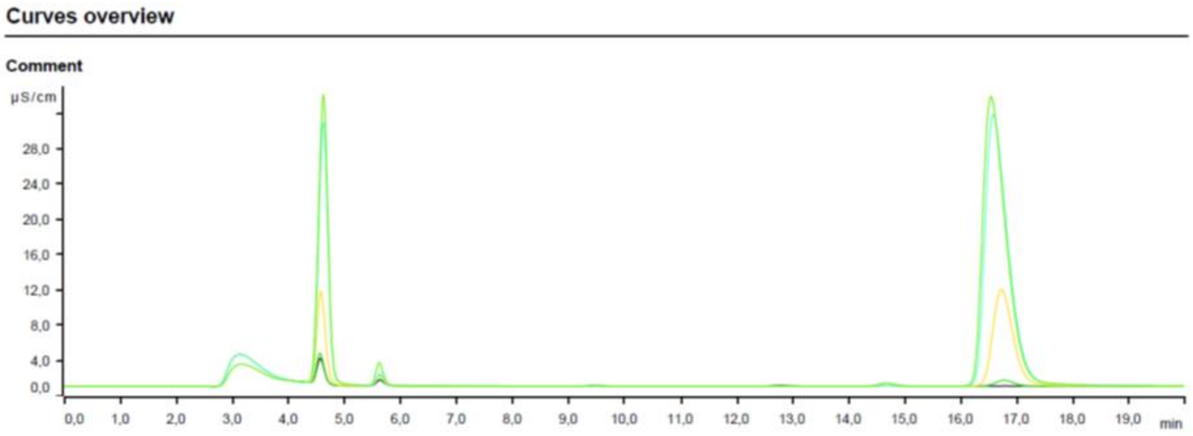

Figure S18. Chromatogram obtained using IC for different calibration standards.

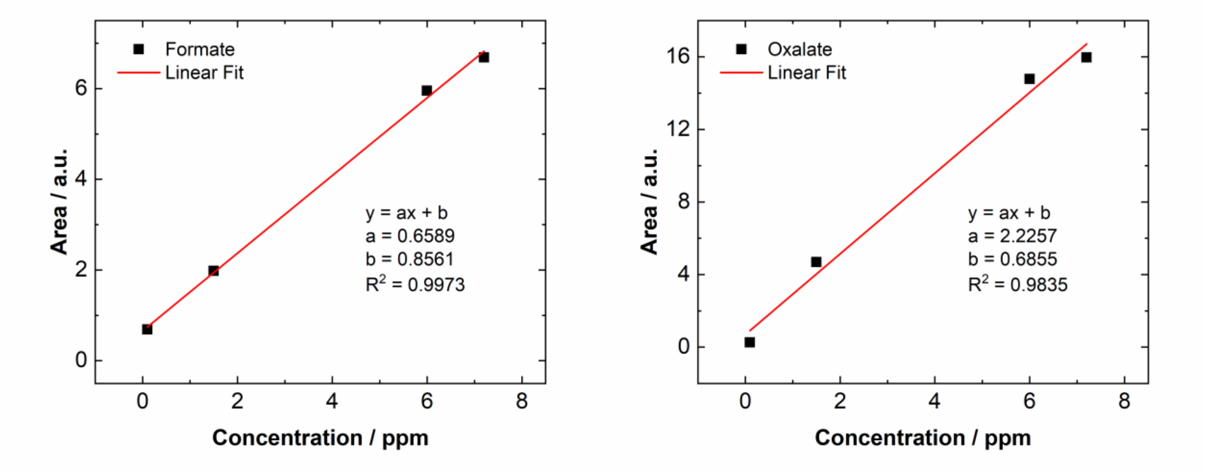

Figure S19. Calibration curves generated for formate and oxalate using IC.

Solution Resistance Estimation

The system in the first experimental configuration also experienced over-voltage trips at -2.3 V (vs. Ag/AgCl), which could be either due to the Pt electrode limiting the oxidation of DMF at the anode side [1] or due to the incorrect compensation of the solution resistance that leads to over-compensation by the potentiostat. The solution resistance for experiments in the first configuration is estimated using a potentiostatic electrochemical impedance spectroscopy (PEIS) at 0.0 V (vs. Ag/AgCl) performed over a frequency range of 100 kHz to 100 mHz. However, with the non-aqueous electrolytes, the PEIS does not produce clear semi-circles and the intersection point of the plot with the x-axis (solution resistance, R_S) is approximately calculated by extrapolating the curve. The estimation of solution resistance (R_S) in the second experimental configuration is performed using the ZIR technique, which essentially performs PEIS at a single high frequency (100 kHz in this case) at 0.0 V (vs. Ag/AgCl) and calculates the resistance value based on the voltammetric response [2]. This technique resulted in a stable current response during the chronoamperometry experiments.

REFERENCES

(1) *Organic Electrochemistry*; Hammerich, O., Speiser, B., Eds.; CRC Press: Boca Raton, 2015.

(2) *Ohmic drop correction: a means of improving measurement accuracy - BioLogic*. https://www.biologic.net/topics/ohmic-drop-correction-a-means-of-improving-measurement-accuracy-with-potentiostats/ (accessed 2022-07-21).
